# Perinatal post-mortem ultrasound (PMUS): radiological-pathological correlation

**DOI:** 10.1186/s13244-019-0762-2

**Published:** 2019-08-21

**Authors:** Susan C. Shelmerdine, Neil J. Sebire, Owen J. Arthurs

**Affiliations:** 1 Great Ormond Street Hospital for Children NHS Foundation Trust, London, WC1N 3JH UK; 2 UCL Great Ormond Street Institute of Child Health, London, UK

**Keywords:** Ultrasound, Autopsy, Diagnostic imaging, Child, Pathology

## Abstract

There has been an increasing demand and interest in post-mortem imaging techniques, either as an adjunct or replacement for the conventional invasive autopsy. Post-mortem ultrasound (PMUS) is easily accessible and more affordable than other cross-sectional imaging modalities and allows visualisation of normal anatomical structures of the brain, thorax and abdomen in perinatal cases. The lack of aeration of post-mortem foetal lungs provides a good sonographic window for assessment of the heart and normal pulmonary lobulation, in contrast to live neonates.

In a previous article within this journal, we published a practical approach to conducting a comprehensive PMUS examination. This covered the basic principles behind why post-mortem imaging is performed, helpful techniques for obtaining optimal PMUS images, and the expected normal post-mortem changes seen in perinatal deaths. In this article, we build upon this by focusing on commonly encountered pathologies on PMUS and compare these to autopsy and other post-mortem imaging modalities.

## Key points

- Post-mortem ultrasound can delineate the main body organs and is primarily used to identify congenital structural anomalies.
- Post-mortem ultrasound is least useful in delineating congenital cardiac anomalies, likely due to the lack of foetal circulation.
- Intracranial pathologies are easily identified, but maceration and overlapping cranial sutures may obscure clear sonographic views.

## Background

Without a perinatal autopsy, many parents cannot understand the cause for their child’s death, clinicians miss detailed information to counsel parents about future pregnancies, and data on epidemiological studies are incomplete [1, 2]. Parents dislike the invasive approach of traditional autopsy, [3], and for others, religious beliefs may preclude an autopsy [4]. The net result of these changes in attitudes has led to an increased parental demand for alternative, non-invasive methods to aid or replace the internal examination component of a perinatal autopsy [3, 5].

Whilst a variety of advanced post-mortem imaging techniques have shown good diagnostic accuracy and reproducibility, such as post-mortem MRI (PMMR) [6], post-mortem CT (PMCT) [7] and post-mortem micro-focus CT (PMμCT) [8], access to these techniques may be restricted especially with limited resources or expertise. Ultrasound is a more affordable, easily accessible and adaptable imaging modality, widely used in many hospital settings. In addition, the ultrasound machinery can be easily transported to various parts of the hospital, avoiding the need to transfer bodies to clinical areas and the disruption that may ensue for other patients and members of staff.

In a recent article published within this journal [9], we described a practical approach to conducting a comprehensive post-mortem ultrasound (PMUS) examination. This covered the basic principles behind foetal preparation for imaging, parental consent, helpful techniques for obtaining optimal PMUS images and the expected normal post-mortem changes.

This current article builds upon our previous work by describing commonly encountered pathologies on PMUS and comparison with results at autopsy and other post-mortem imaging modalities, where available. We intend this to serve as a useful pictorial reference for radiologists, clinicians and allied healthcare professionals who wish to start providing a perinatal PMUS service.

## Pre-imaging considerations

Whilst care and thoroughness should be exercised when imaging foetuses irrespective of their prenatal history, it is important to understand the type of suspected pathology to be excluded and the clinical significance which should be placed on incidental findings. Three key considerations should therefore be taken into account—gestation, the prenatal imaging findings and the mode of foetal demise (i.e. termination of pregnancy, stillbirth or miscarriage). Gestational-dependent changes associated with foetal developmental are well recognised and not reproduced here.

Prenatal ultrasound screening is now commonplace in many developed nations, and modern imaging techniques have a high diagnostic accuracy rate in the region of 90% when compared with subsequent perinatal autopsy findings [10, 11]. It is therefore important to support or dispute prenatal diagnoses where possible with post-mortem examinations. Particular care should be taken when assessing prenatally diagnosed abdominal or musculoskeletal pathologies, as several studies have reported a lower prenatal ultrasound diagnostic accuracy rate for these body systems: approximately 60% for intra-abdominal pathologies [10, 12], and < 30% for limb anomalies [12]. In one study of over 500 foetuses, the highest false-positive rates were given for gastrointestinal and renal tract malformations [13].

Regarding mode of foetal demise, a history of termination of pregnancy is particularly pertinent. This is because PMUS is able to identify congenital structural abnormalities (but less able to diagnose infection or placental aetiologies) and a high number of terminations of pregnancy will be due to antenatally detected structural abnormalities (estimated at approximately 43.8 [14] to 85.8% [15] compared to only 5% of intra-uterine deaths and stillbirths [16]). Where a clinical indication states that a termination of pregnancy has occurred, the radiologist should be aware of the reasons behind this decision and seek to examine carefully the organs that had been suspected of significant anomalies.

### Diagnostic accuracy

Large surveys of parents and healthcare professionals have found that whilst imaging techniques for non-invasive autopsies have a high acceptability [5, 17], one barrier to uptake are concerns about ‘missed diagnoses’, potentially not reaching the same levels of certainty as an ‘invasive’ autopsy [18]. Whilst PMMR has a more established role and its usage is widely published [7, 19–24], compared to conventional perinatal autopsy findings, the diagnostic accuracy of PMUS is still being evaluated.

Overall PMUS sensitivity and specificity rates have been reported at 75% and 83.3% for whole-body diagnoses [25], with abnormalities affecting the heart being particularly challenging to diagnose with lower sensitivity rates ranging between 18.2–50% [25–27]. These numbers may be lower than expected or acceptable, although when comparing 2-D PMUS and 3T PMMR imaging within the same cohort of 160 foetuses, Kang et al. [28] did not find a statistically significant difference in accuracy rates for cases where the ultrasound images were of diagnostic quality (67.8% vs 78.0% respectively, *p* > 0.05). This suggests that a well-performed perinatal post-mortem ultrasound study could provide a similar degree of information as PMMR without the need for further cross-sectional imaging, allowing for potential placement as a first-line technique to triage cases for PMMR. In this article, cases where PMUS was found to be non-diagnostic (i.e. unhelpful) were those that were performed in foetuses of < 20 weeks gestational age [28] and displaying moderate or severe maceration [29]. Therefore, we recommend that operators are aware of the reduced likelihood of a diagnostic PMUS study in small gestational aged foetuses, those that have a known prolonged intra-uterine retention period and those with suspected cardiac anomalies.

## Systems review

Our approach to a comprehensive PMUS is based on a structured ‘systems review’ of the foetus. This enables a review of the main body organs for congenital anatomical variants or abnormalities. In this section, we demonstrate some of these major pathologies by body systems and describe their relative importance.

### Head, neck and spine

Congenital disorders of the central nervous system (CNS) are the commonest group of structural anomalies leading to termination of pregnancy. Approximately 32–37.7% of all terminations of pregnancies may be for prenatally diagnosed CNS anomalies [14, 30], with neural tube defects accounting for the largest proportion (approximately a third of cases) [31].

**Fig. 1:**
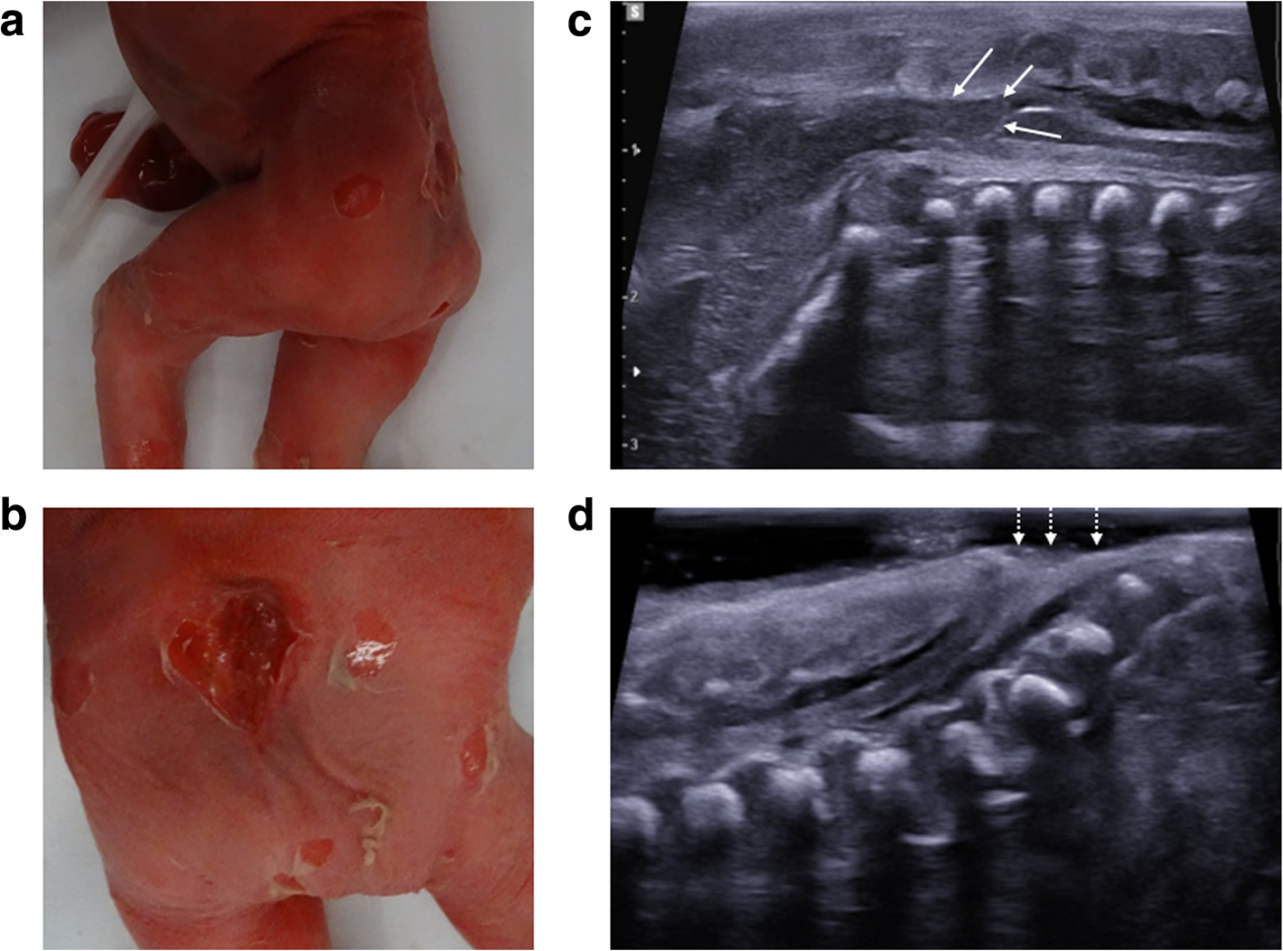
Neural tube defect following termination of pregnancy in a foetus at 25 weeks gestation. A small neural tube defect is identified on external examination at autopsy with the foetus viewed from the left side (**a**) and from behind (**b**). On the corresponding post-mortem ultrasound performed 4 days after death, the sagittal view of the cranio-cervical junction (**c**) demonstrates herniation of the cerebellar tonsils (solid white arrows) into the upper cervical spinal canal. The sagittal ultrasound image of the lumbosacral spine (**d**) demonstrates the small neural tube defect (dashed arrows)

**Fig. 2:**
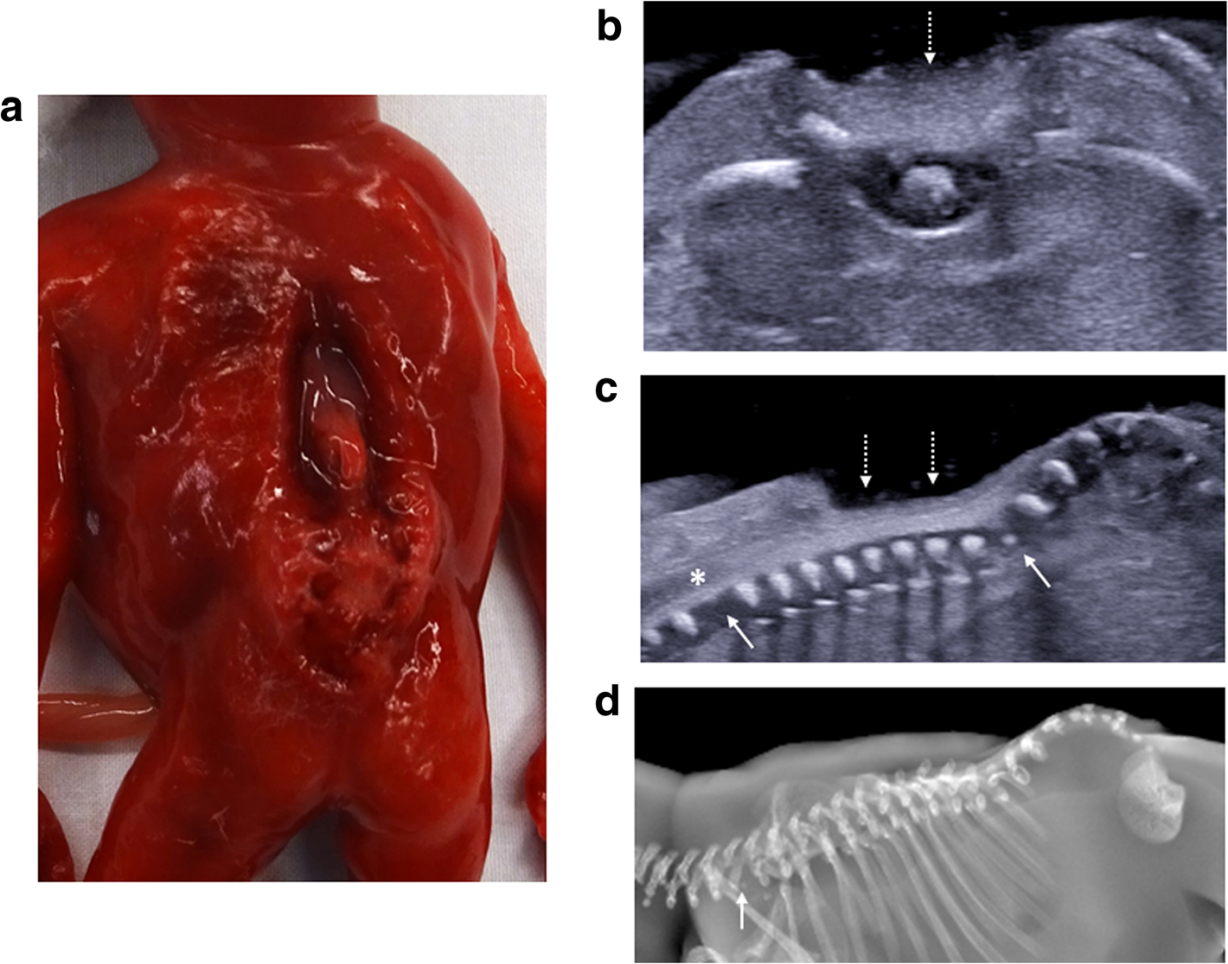
Neural tube defect following termination of pregnancy in a foetus at 16 weeks gestation. On external examination at autopsy (**a**), the large neural tube defect is readily appreciated. The corresponding post-mortem ultrasound images were obtained 4 days after death in transverse (**b**) and sagittal (**c**) planes, demonstrating the absence of posterior vertebral elements (dotted arrows), irregular thoracolumbar vertebral ossification (solid arrows) and high conus medullaris of the spinal cord (asterisk). The lateral post-mortem radiograph of the spine (**d**) was able to demonstrate sacral spinal dysraphism and irregular thoracolumbar vertebral ossification (solid arrow), although these findings were better depicted on ultrasound

For this reason, it is important to assess the spine in all foetuses, paying close attention for associated Chiari malformations (Fig. 1) and identifying vertebral segmental anomalies and spinal dysraphism (Fig. 2). Further characterisation with skeletal radiographs may be helpful [32].

**Fig. 3:**
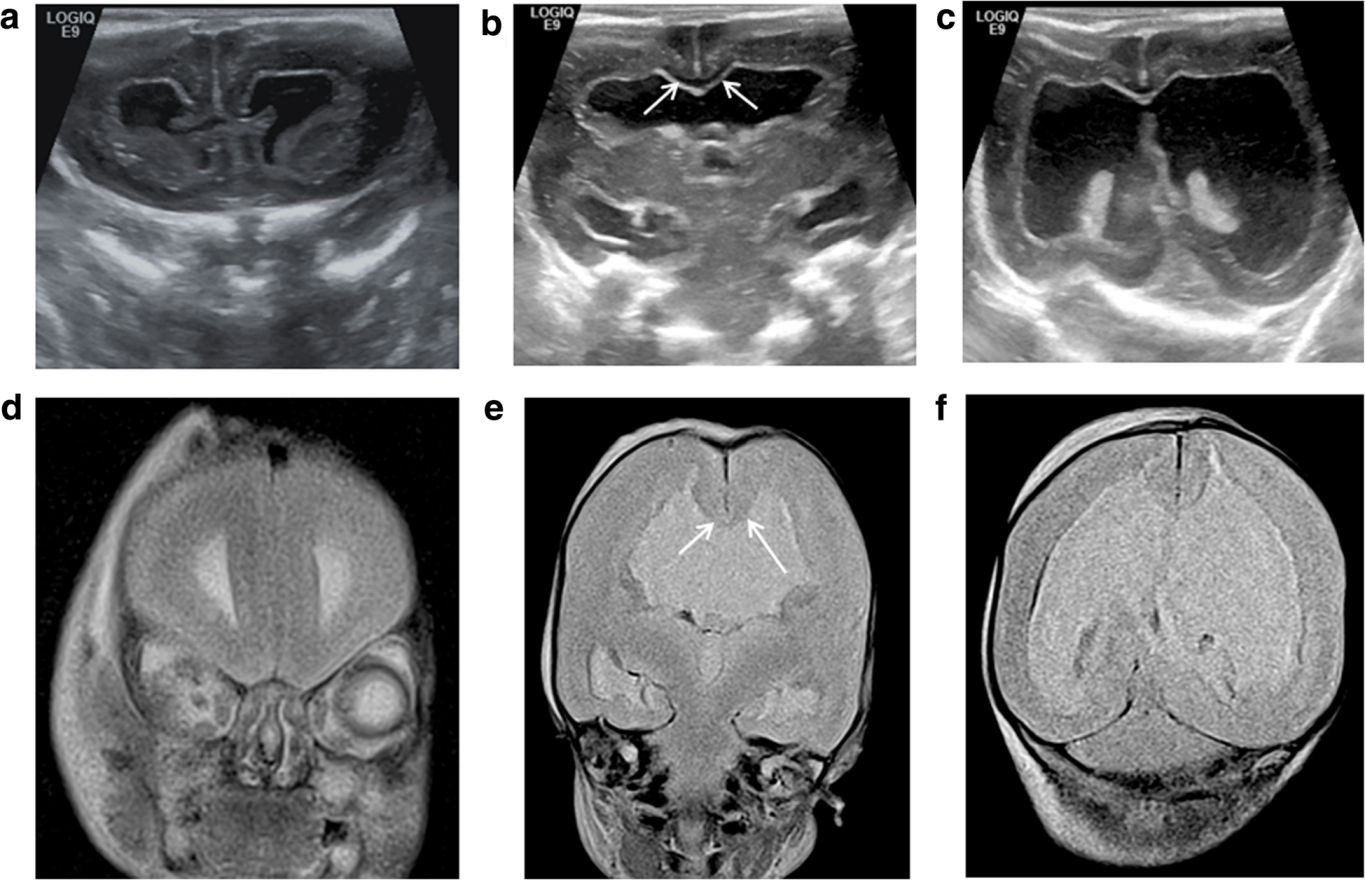
Post-mortem ultrasound images of the brain, in coronal section (top row), with matched T2-weighted post-mortem MRI images (bottom row) performed on the same day, in a foetus at 21 weeks gestation, 9 days after death. These have been obtained through the frontal lobe (**a**, **d**), at the level of the Foramen of Monro (**b**, **e**) and through the posterior horns of the lateral ventricles (**c**, **f**). The pregnancy was terminated for suspected foetal ventriculomegaly and an absent corpus callosum. From the post-mortem ultrasound, the ventriculomegaly was well depicted before the MRI was performed, and a corpus callosum was in fact present (white arrows)

**Fig. 4:**
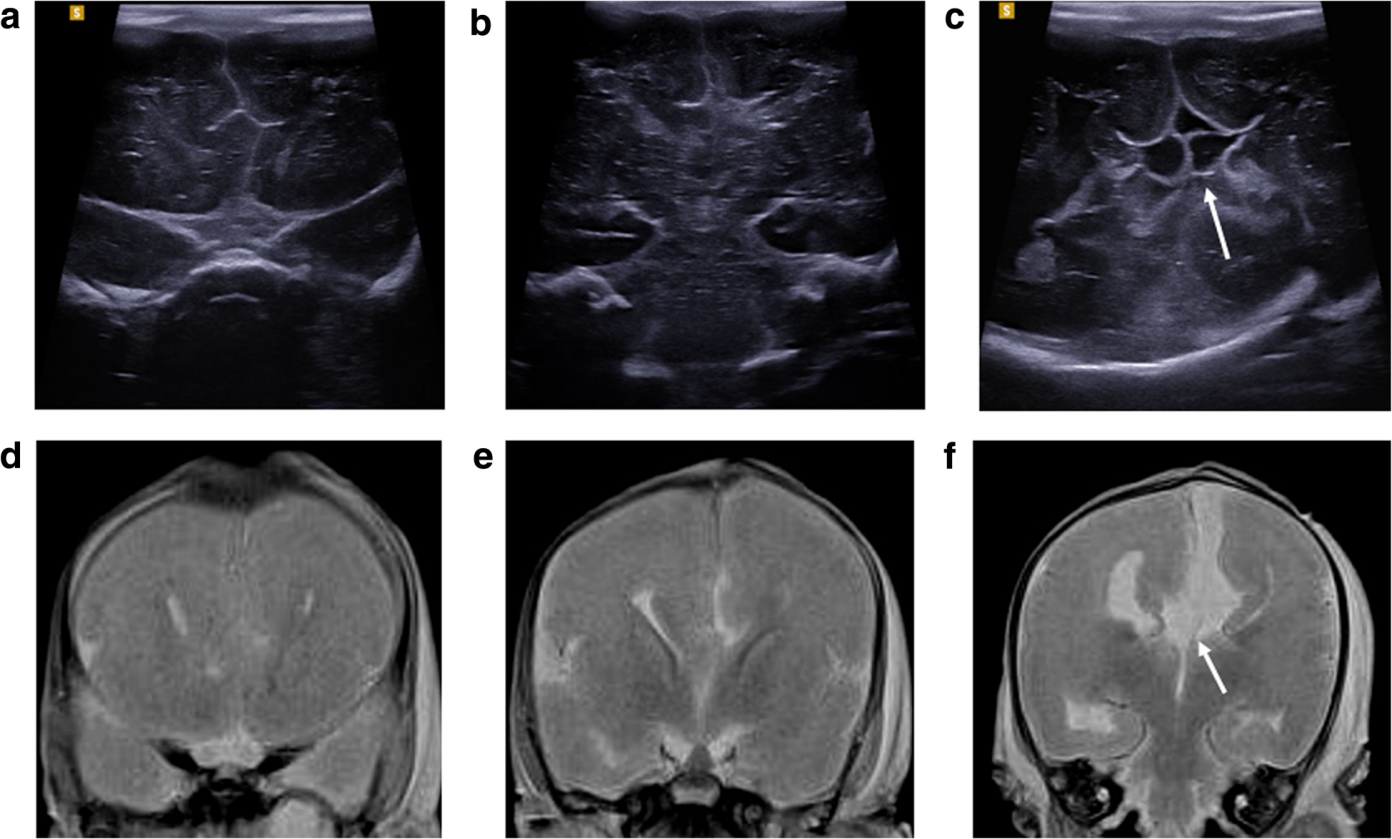
Post-mortem ultrasound images of the brain, in coronal section (top row), with matched T2-weighted post-mortem MRI images (bottom row) in a foetus at 25 weeks gestation. The pregnancy was terminated for suspected brain anomalies. Both imaging modalities were performed 2 days after delivery. The images demonstrate views through the frontal lobes (**a**, **d**), at the level of the Foramen of Monroe (**b**, **e**) and through the posterior horns of the lateral ventricles (**c**, **f**). The ultrasound image clearly depicts an interhemispheric cyst (white arrow) with internal septations (**c**), and there is absence of the corpus callosum. This is also evident from the MRI image (**f**), although the cyst is much better viewed on ultrasound

**Fig. 5:**
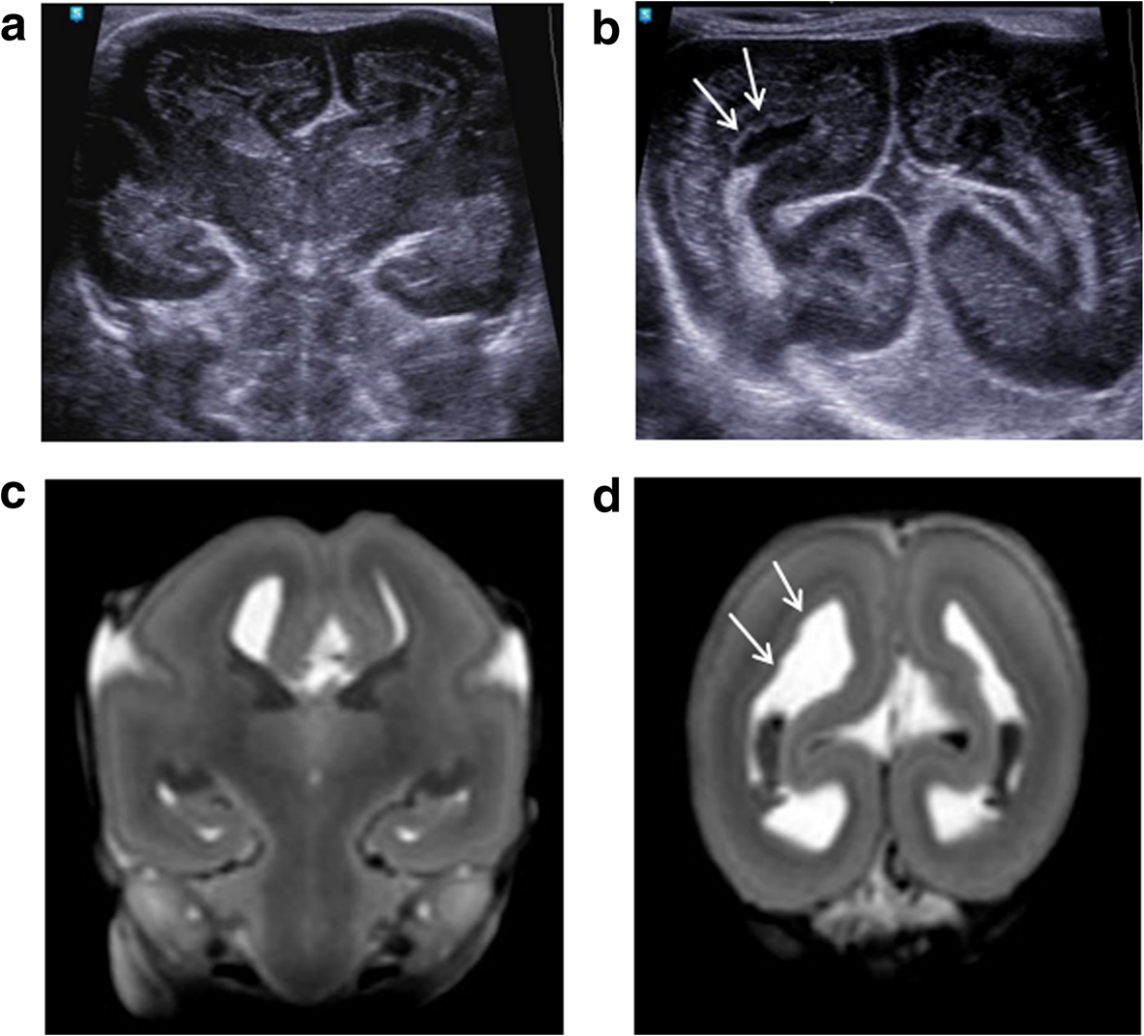
Post-mortem ultrasound images of the brain, in coronal section (top row), with matched T2-weighted post-mortem MRI images (bottom row) in a foetus at 18 weeks gestation. Termination of pregnancy was performed for suspected brain anomalies. Both imaging modalities were performed on the same day, 7 days after death. The images demonstrate views through the Foramen of Monroe (**a**, **c**) and through the posterior horns of the lateral ventricles (**b**, **d**). From both imaging modalities, there is moderate ventriculomegaly, with subtle irregularity of the ependymal lining best seen along the posterior horns of the lateral ventricles. This suggests an underlying neuronal migration defect, subsequently confirmed at autopsy

The second most common CNS anomaly is lateral ventricular dilatation, or ventriculomegaly. Where prenatal ventricular dilatation is reported, cerebrospinal fluid shifts occurring after death may result in an apparent ‘resolution’ of this appearance [33]. In those instances, PMUS cannot confirm nor refute ventriculomegaly per se but should be used to exclude other contemporaneous anomalies [34] such as callosal anomalies (Figs. 3 and 4), neuronal migration anomalies (Fig. 5) or cerebral aqueductal stenosis, although the latter is harder to identify at post-mortem in the presence of cerebral oedema and sutural distortion.

**Fig. 6:**
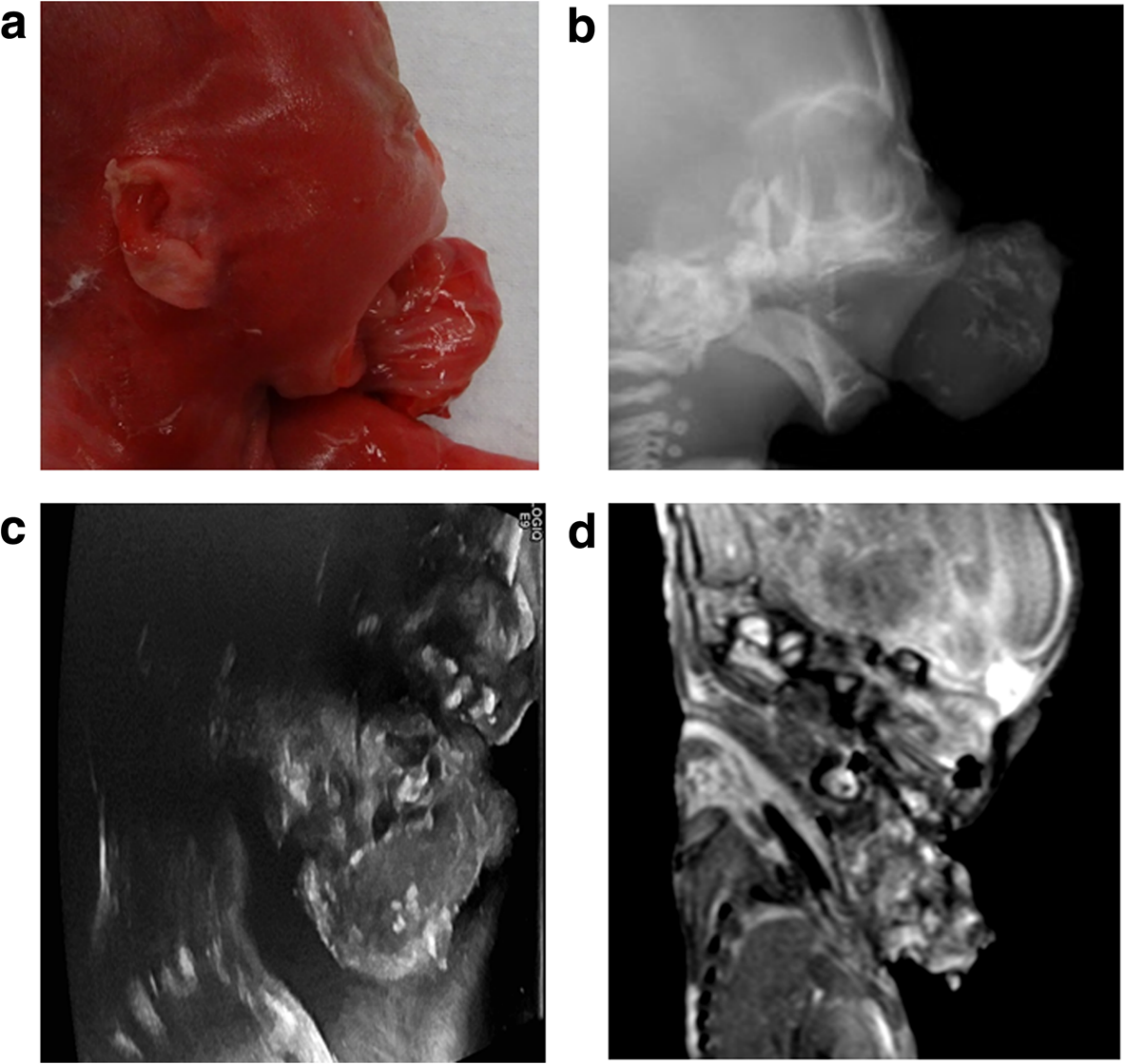
Epignathus arising from the hard palate in a foetus at 21 weeks gestation, after a termination of pregnancy for suspected anterior encephalocele. The photograph taken prior to autopsy of the head (**a**) demonstrates a large soft tissue mass arising from the oral cavity. The corresponding lateral radiograph of the head (**b**) demonstrates multiple calcific components within. A sagittal view during post-mortem ultrasound (**c**) and post-mortem MRI (**d**) obtained 7 days after delivery, both demonstrate the heterogenous internal nature of the oral mass, although it is much harder to identify the origin of the mass on the PMMR given the position, resolution and slumping of the foetus in the scanner

**Fig. 7:**
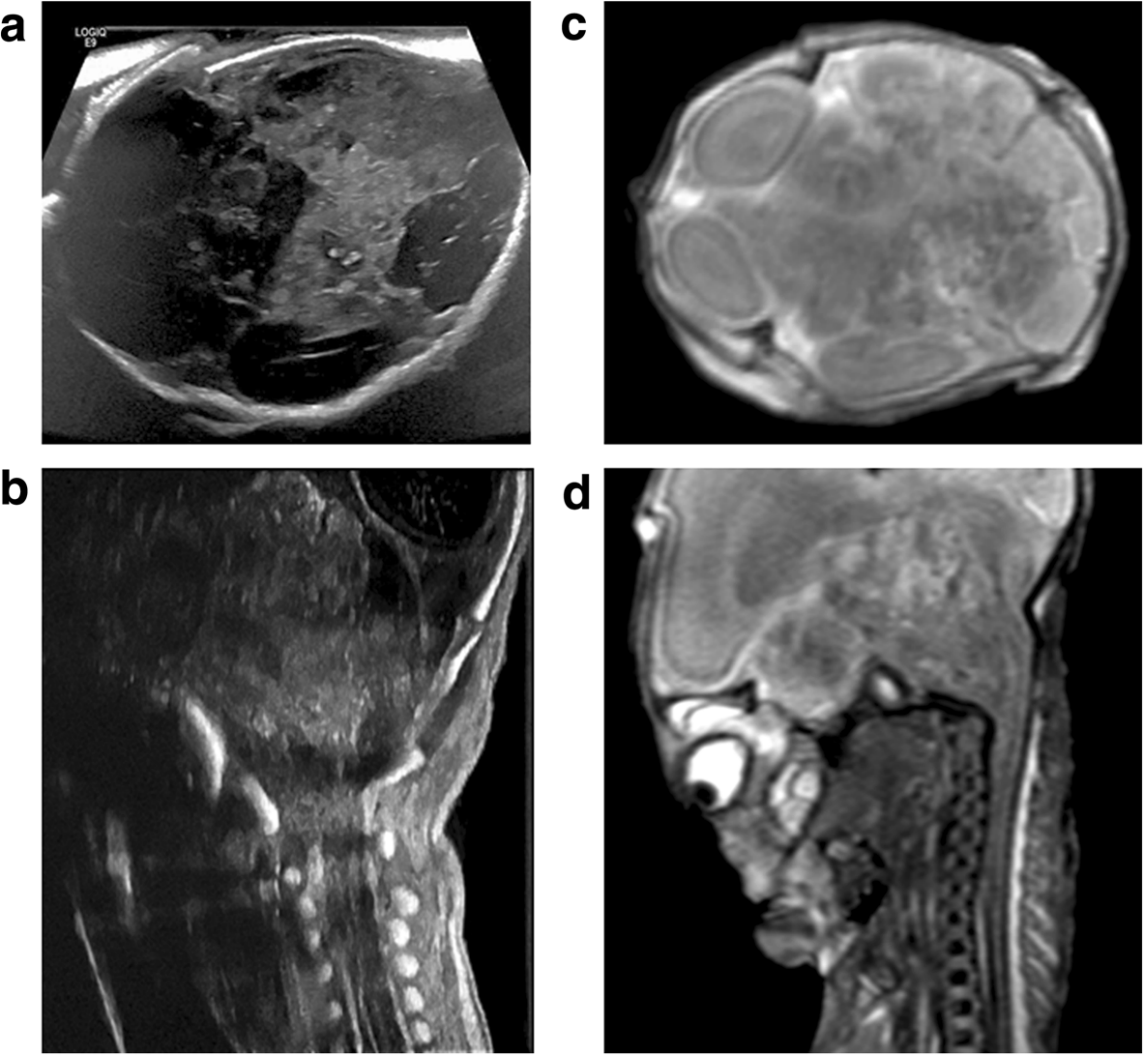
In the same foetus at 21 weeks gestation, described in Fig. 6, there were several intracranial abnormalities. Post-mortem ultrasound images of the brain in axial plane (**a**) and the cervical spine in sagittal plane (**b**) are matched with corresponding T2-weighted post-mortem MRI images (**c**, **d**). Both imaging modalities were acquired 7 days after delivery. This infiltrating intracranial mass affecting the brainstem and both temporal lobes was also a teratoma. The lesion is mostly echogenic on ultrasound but of heterogenous signal intensity on MRI

**Fig. 8:**
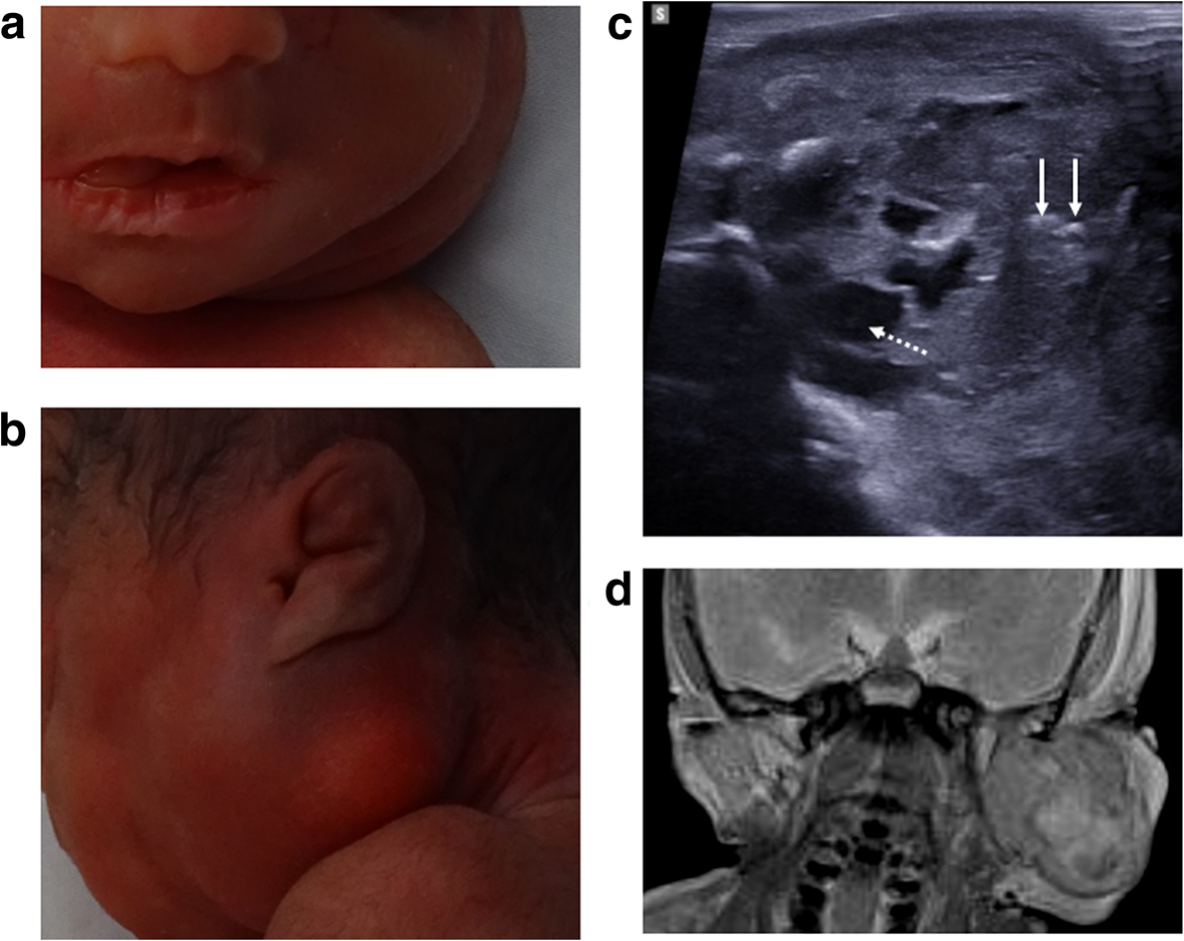
Post-mortem images of a foetus at 25 weeks gestation with a prenatally detected neck mass. Post-mortem photographs at external examination from the front (**a**) and of the left side of the neck (**b**) demonstrate the soft tissue neck mass. The post-mortem ultrasound (**c**) was performed 2 days after delivery and demonstrated mixed solid and cystic elements with some internal calcific foci in this lesion. The subsequent post-mortem MRI (**d**) performed on the same day confirms the ultrasound findings of a solid and cystic mass, although it was harder to demonstrate the internal calcific foci. At histopathological assessment, the mass was found to represent a teratoma

**Fig. 9:**
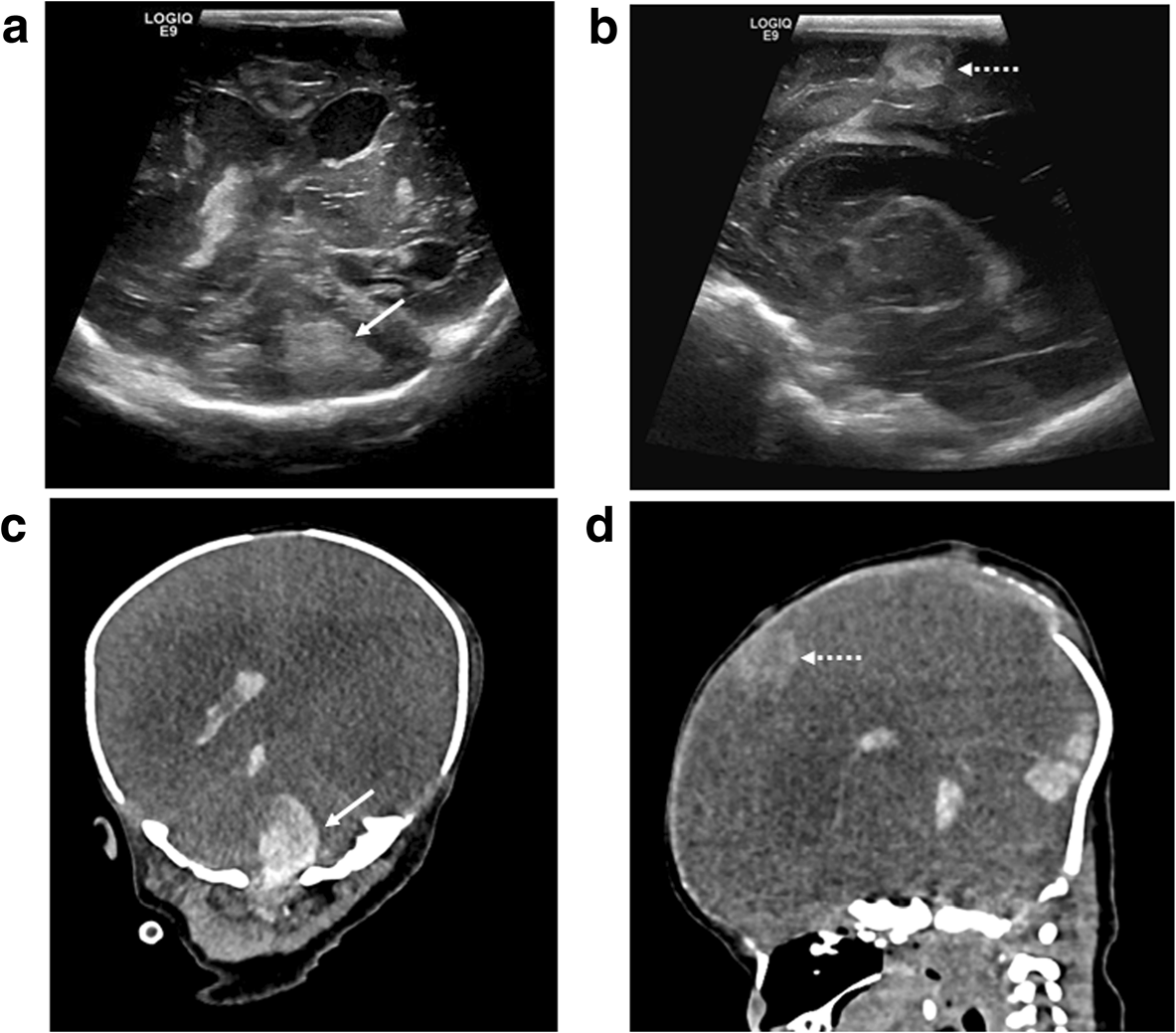
Post-mortem cranial ultrasound images (top row), with matched unenhanced post-mortem CT images (bottom row) of a fourteen day old term neonate who died from a metastatic fibrosarcoma affecting the right thigh. Both imaging modalities were performed 3 days after death. The coronal (**a**, **c**) and sagittal (**b**, **d**) images demonstrate bilateral lateral ventricular dilatation with several hyperechoic and hyperdense metastatic deposits within the cerebellum (white arrows) and along the falx (dashed arrow). There is loss of grey white matter differentiation with poorly defined ventricles on the CT compared to ultrasound, given the significant underlying cerebral oedema

Congenital intracranial tumours are rare. These account for < 2% of all foetal tumours [35], and teratomas forming the vast majority of all subtypes (Figs. 6 and 7). As a group, congenital intracranial tumours have a poor survival rate (estimated in one study as only 7% in the first year of life [36]), and tumours arising from or extending into the neck are usually at high risk of airway obstruction during delivery (Fig. 8). Intracranial lesions are usually primary tumours rather than metastases, although we have imaged one neonate with multiple intracranial metastases from a congenital fibrosarcoma of the thigh (Fig. 9). Recurrence rates in future pregnancies for intracranial anomalies are fortunately low, particularly where the abnormality is isolated (which is the case for the majority) and an underlying genetic cause is not identified [37].

### Cardiac and vascular imaging

Congenital cardiac anomalies make up a significant proportion (approximately 10% [31]) of all structural anomalies at termination of pregnancy, and the prevalence of such anomalies are rising. Over the last 20 years across Europe, the annual increase of cardiac anomalies has been estimated at 1.4–4.6% [38] with the reasons thought to be related to increased maternal risk factors such as diabetes, body mass index, assisted reproductive techniques and alcohol consumption. These are key aspects of the mother’s clinical history which should be available at post-mortem imaging.

At PMUS the detection of complex cardiac anomalies is difficult due to a combination of lack of circulating blood, intra-cardiac haemostasis and occasionally intra-cardiac gas (likely from feticide [39]). Some distortion of the normal anatomy at post-mortem examination can be overcome by imaging the foetus in a waterbath [9].

**Fig. 10:**
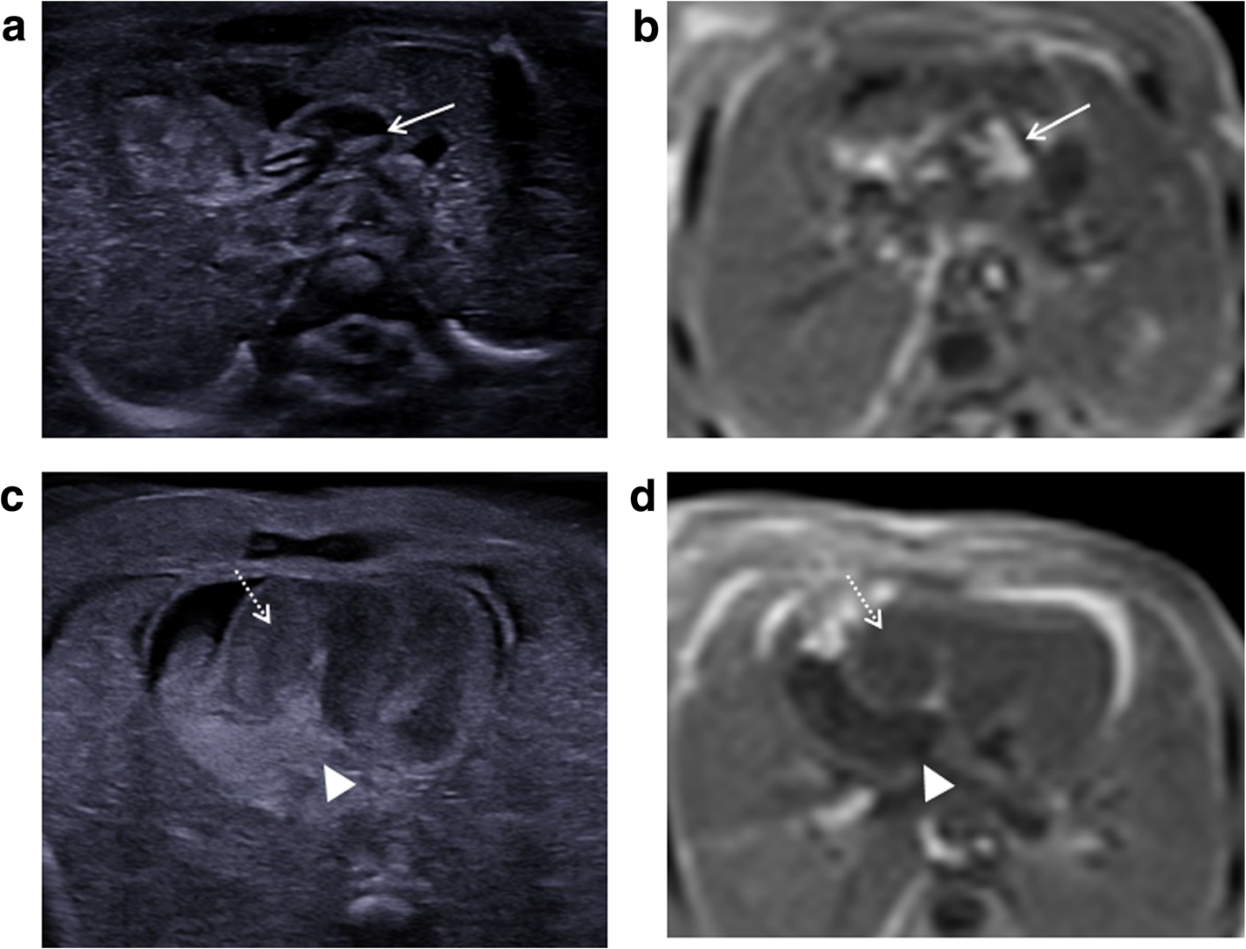
Supravalvular pulmonary artery stenosis in a foetus at 21 weeks gestation. The imaging was performed 5 days after termination of pregnancy. Transverse images of the outflow tracts on post-mortem ultrasound (**a**) and T2 weighted MRI at 1.5T (**b**) both demonstrate a dilated main pulmonary artery (solid arrows). The transverse four cardiac chamber view on ultrasound (**c**) and MRI (**d**) show a dilated and thick-walled right ventricle (dotted arrow) and small atrial septal defect (arrowhead). Given the small size of the foetus, the findings were more easily appreciated on ultrasound than MRI

**Fig. 11:**
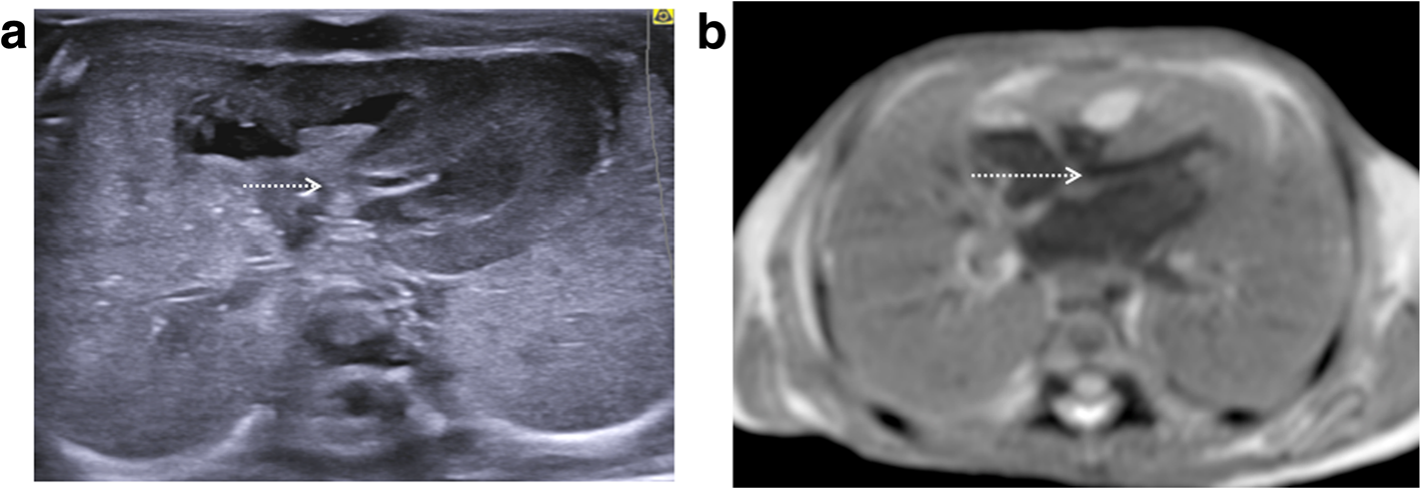
Paired transverse images of the four cardiac chambers in a foetus at 19 weeks gestation, obtained 6 days after termination of pregnancy. Post-mortem ultrasound image (**a**) and T2-weighted MRI at 1.5T (**b**) both show a small ventricular septal defect (dotted white arrow). It is also interesting to note the normal ‘expected’ post-mortem intra-cardiac clot (echogenic material on ultrasound, low signal on MRI) and fluid (hypoechoic on ultrasound, high signal on MRI)

The commonest cardiac anomalies at termination of pregnancy are hypoplastic right/left heart syndrome [31] and uni-ventricular heart defects [40]. Other pathologies also feature, although less commonly, and include pulmonary atresia/stenosis (Fig. 10), aortic valve atresia/stenosis, transposition of the great arteries, tetralogy of Fallot, coarctation of the aorta, anomalous pulmonary venous return and septal defects (Fig. 11).

Where cardiac imaging is non-diagnostic at PMUS, further cross-sectional imaging with PMMR may be useful, particularly if high-resolution, isovolumetric sequences for multiplanar reconstructions are acquired, given the post-mortem distortion of normal anatomy due to ‘slumping’.

### Thorax

Congenital pulmonary anomalies are the least common structural abnormalities seen at PMUS [25]. Whilst congenital pulmonary malformations (including cystic malformations, bronchopulmonary sequestrations, bronchial atresia, congenital lobar emphysema and bronchogenic cysts) may all be seen in live children, these are rarely the cause for foetal demise or terminations of pregnancy [41, 42]. In our experience, we have not detected any airway or lung malformations on PMUS, although Kang et al. [25] report one autopsy confirmed case of a bronchopulmonary foregut malformation in their series, which was missed on PMUS. It could have been due to the subtlety of the appearances that lead to the miss on PMUS; however, the medical literature is sparse with regards to the ideal post-mortem imaging of congenital pulmonary malformations.

**Fig. 12:**
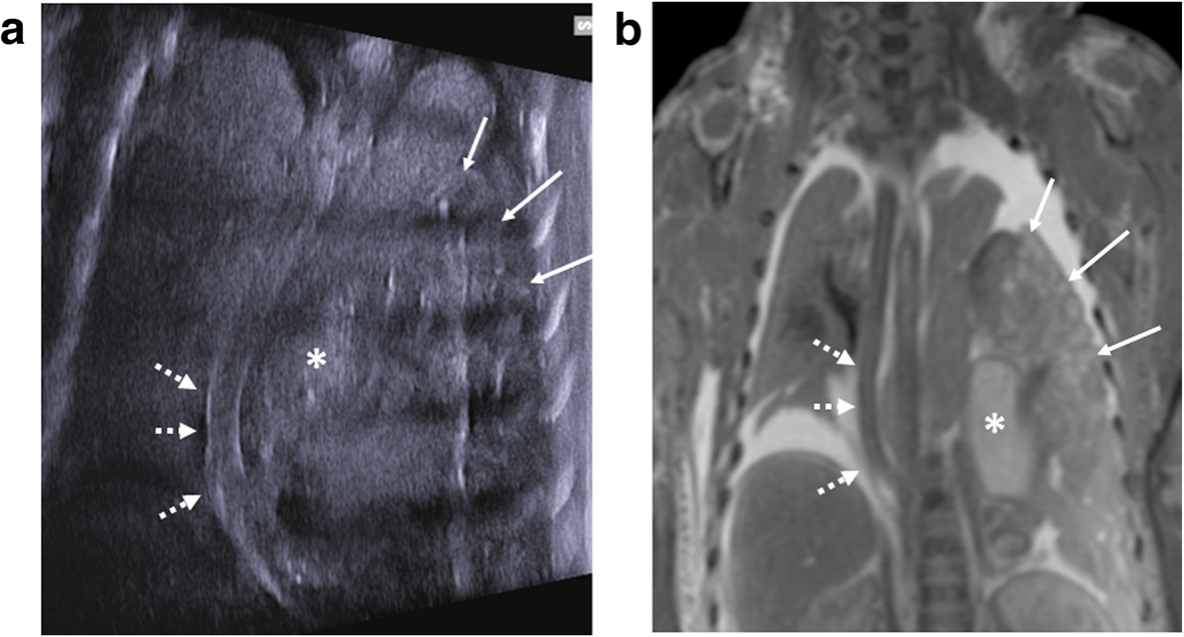
Large left sided congenital diaphragmatic hernia in a foetus at 24 weeks gestation, following termination of pregnancy. Post-mortem ultrasound and MRI were performed 24 hours apart, 12 days after death. Coronal plane imaging of the thorax at post-mortem ultrasound (**a**) and post-mortem T2 weighted MRI at 1.5T (**b**) show herniated bowel loops (solid white arrows), herniated stomach (asterisk) and the deviated course of the oesophagus (dotted white arrows)

The commonest finding at post-mortem imaging of the lungs is lung hypoplasia, usually secondary to other intra-abdominal pathologies such as congenital diaphragmatic hernias (Fig. 12) or enlarged polycystic kidneys. Excluding pulmonary infection is not currently possible [9] given that the foetal and early neonatal lungs are normally fluid filled.

### Abdomen

**Fig. 13:**
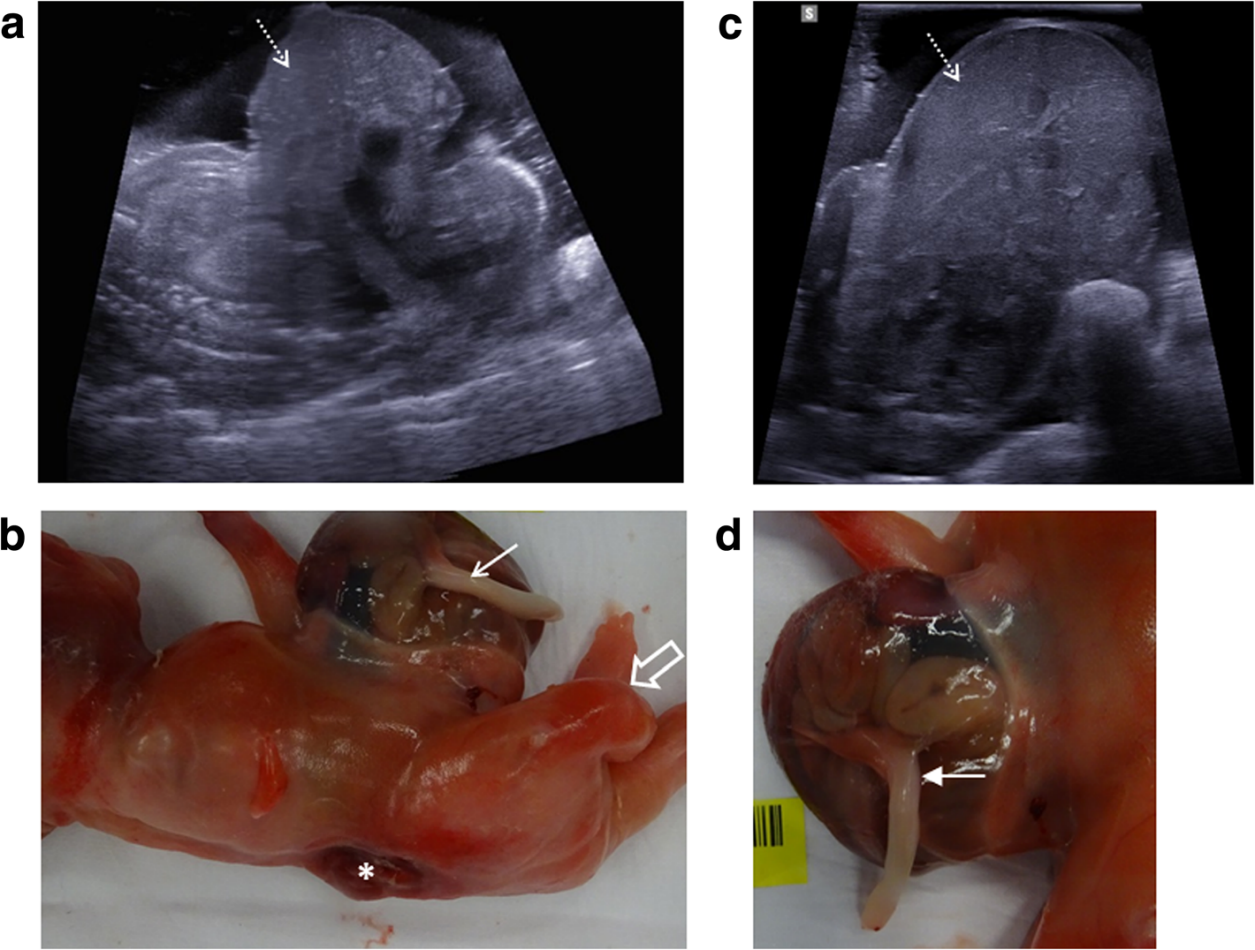
Large exomphalos in a foetus at 22 weeks gestation, following termination of pregnancy for multiple congenital anomalies. Post-mortem ultrasound performed 10 days after delivery in both sagittal (**a**) and transverse plane (**c**) demonstrate herniation of bowel contents through the anterior abdomen. The contents of the exomphalos contained loops of bowel and the majority of the liver (dotted arrow). The external photographs of the foetus at autopsy (**b**, **d**) show the herniated contents being covered by a thin membrane with the umbilical cord arising from this (solid arrow). It is also possible to identify further anomalies of a neural tube defect (asterisk) and hypoplastic right limb (unfilled arrow) from the external images

Abnormalities of the abdomen seen at PMUS are most commonly related to the urinary tract or abdominal wall, the latter including pathologies such as gastroschisis, omphalocele (Fig. 13) and congenital diaphragmatic hernia (Fig. 12) [25, 26, 43]. Whilst the presence of an anterior abdominal wall defect does not require ultrasound for diagnosis, the resultant distortion and shift of intra-abdominal organs may have made prenatal imaging difficult and therefore examination of the presence of internal structures is the main criteria for imaging these cases.

**Fig. 14:**
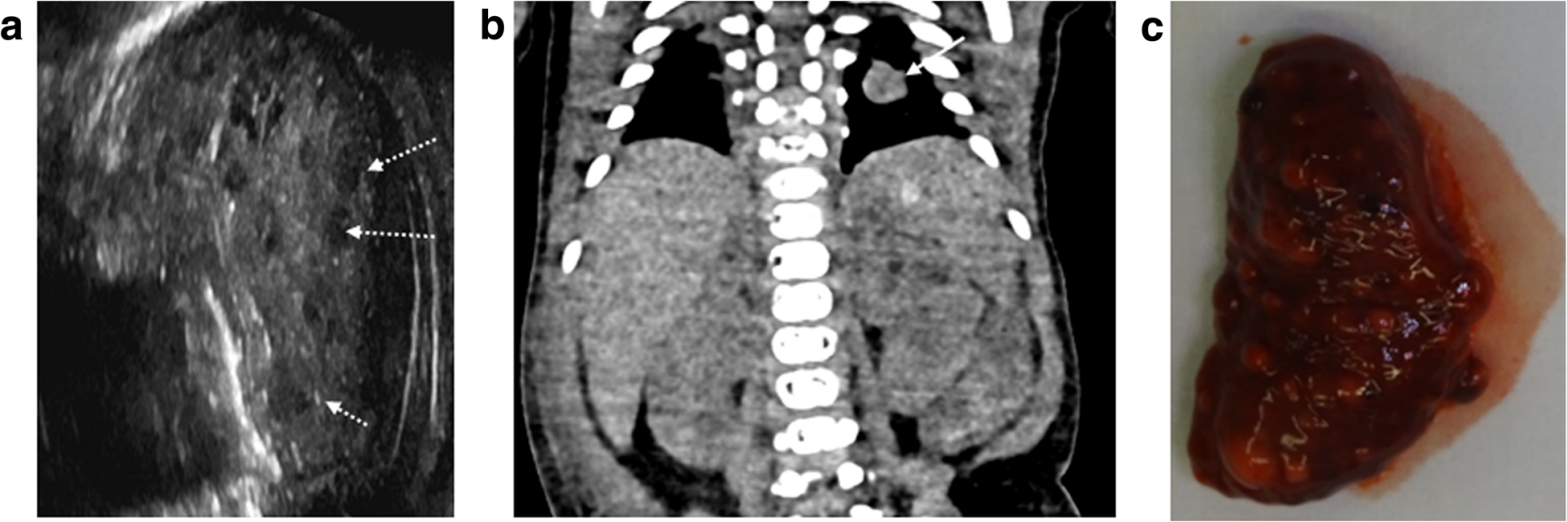
**a**–**c** Multiple splenic metastases from infantile fibrosarcoma in a 14 day old neonate (same case as in Fig. 9). Post-mortem imaging was performed 3 days after death. The post-mortem ultrasound in coronal section (**a**) demonstrates numerous hypoechoic lesions throughout the spleen (dotted arrows). On the corresponding coronal unenhanced CT image of the upper abdomen (**b**), the splenic lesions are less apparent, although a pulmonary metastasis in the left lung (solid arrow) is noted. At autopsy, the extracted spleen (**c**) reveals numerous solid lesions

**Fig. 15:**
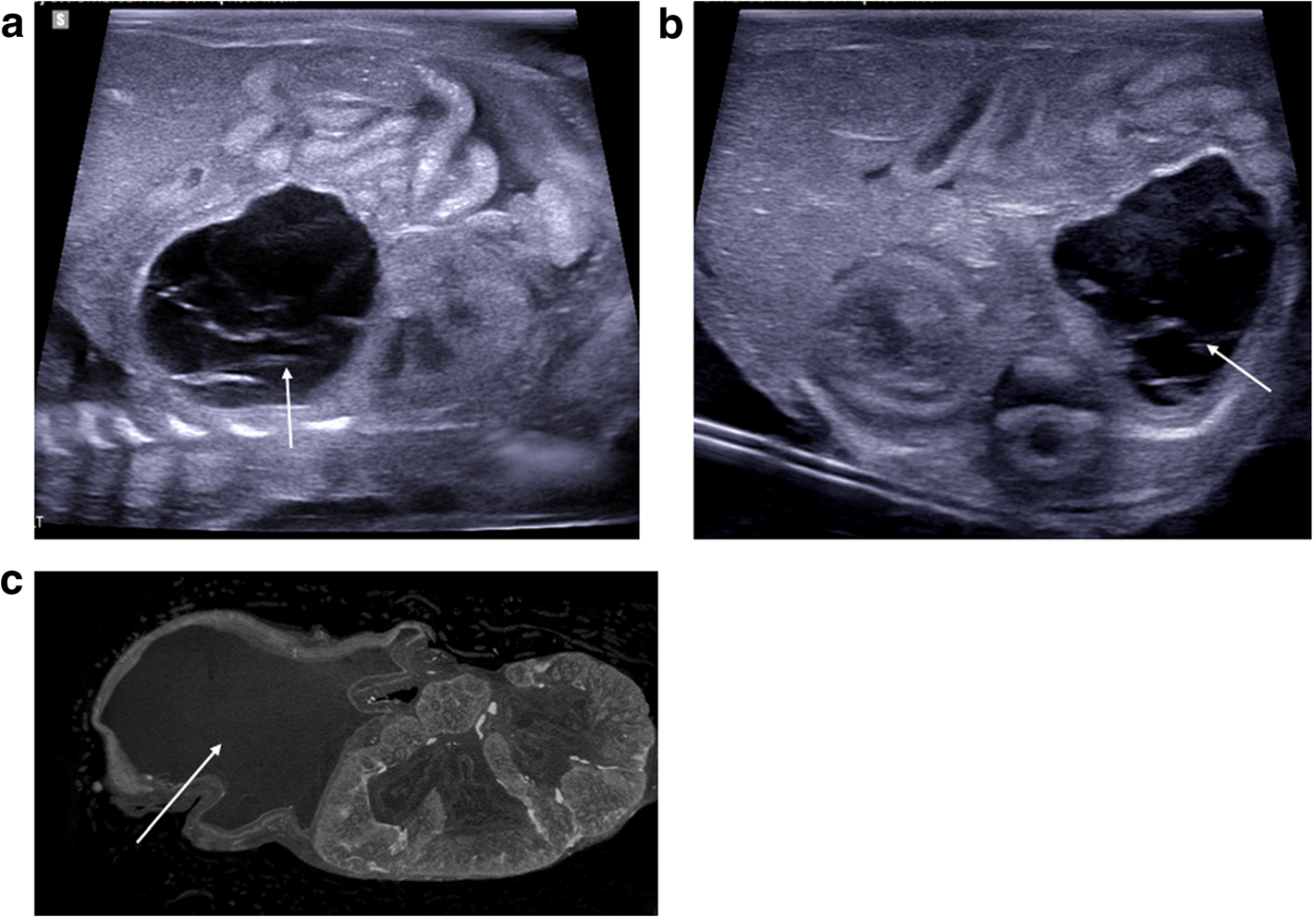
Left adrenal haemorrhage in a foetus at 18 weeks gestation, following termination of pregnancy for megalourethra. The post-mortem ultrasound images of the left kidney in sagittal section (**a**) and of the adrenal glands in transverse plane (**b**) were obtained 6 days after delivery. There is a large septated, cystic mass in the left suprarenal space (solid arrows), better depicted on the sagittal contrast enhanced micro-CT imaging (**c**) of the extracted left kidney. The findings were in keeping with a small haematoma from in utero adrenal haemorrhage

Congenital intra-abdominal foetal tumours are very rare but may occur in the liver (such as haemangiomas, mesenchymal hamartoma and hepatoblastomas), kidneys (mesoblastic nephroma), pelvis (sacrococcygeal teratoma) or adrenal gland (neuroblastoma) [44]. We have previously identified splenic metastases from an aggressive primary fibrosarcoma (Fig. 14) and a suprarenal cystic mass secondary to in utero adrenal haemorrhage (Fig. 15).

**Fig. 16:**
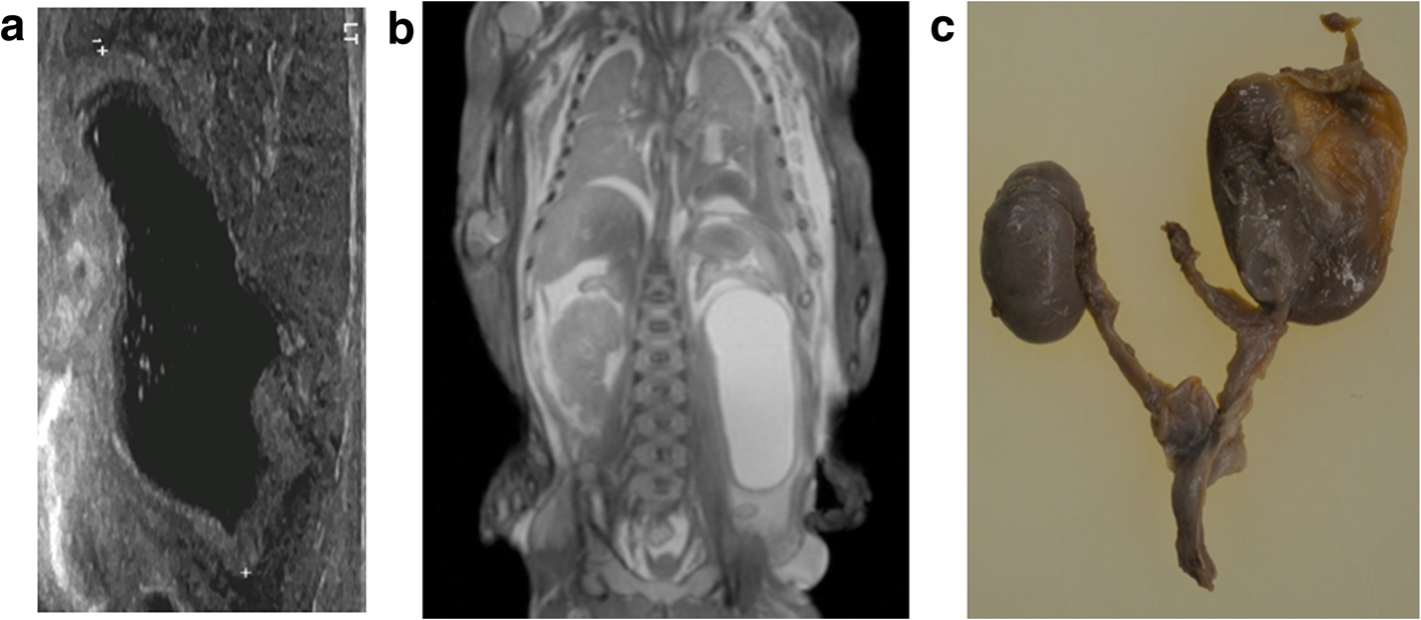
Unilateral left renal outflow obstruction from pelviureteric junction obstruction (PUJO) in a foetus at 21 weeks gestation, after termination of pregnancy. Post-mortem imaging was performed 10 days after delivery. The sagittal post-mortem ultrasound image of the left kidney (**a**) demonstrates a dilated renal pelvis, with only a thin rind of renal cortex stretched around the pelvis. The same finding is shown on the post-mortem T2-weighted MRI image in coronal plane (**b**), and a normal right kidney is demonstrated. A photograph of the extracted kidneys and ureters at autopsy (**c**) confirm the imaging findings of a much larger and dilated left kidney, with normal right kidney

**Fig. 17:**
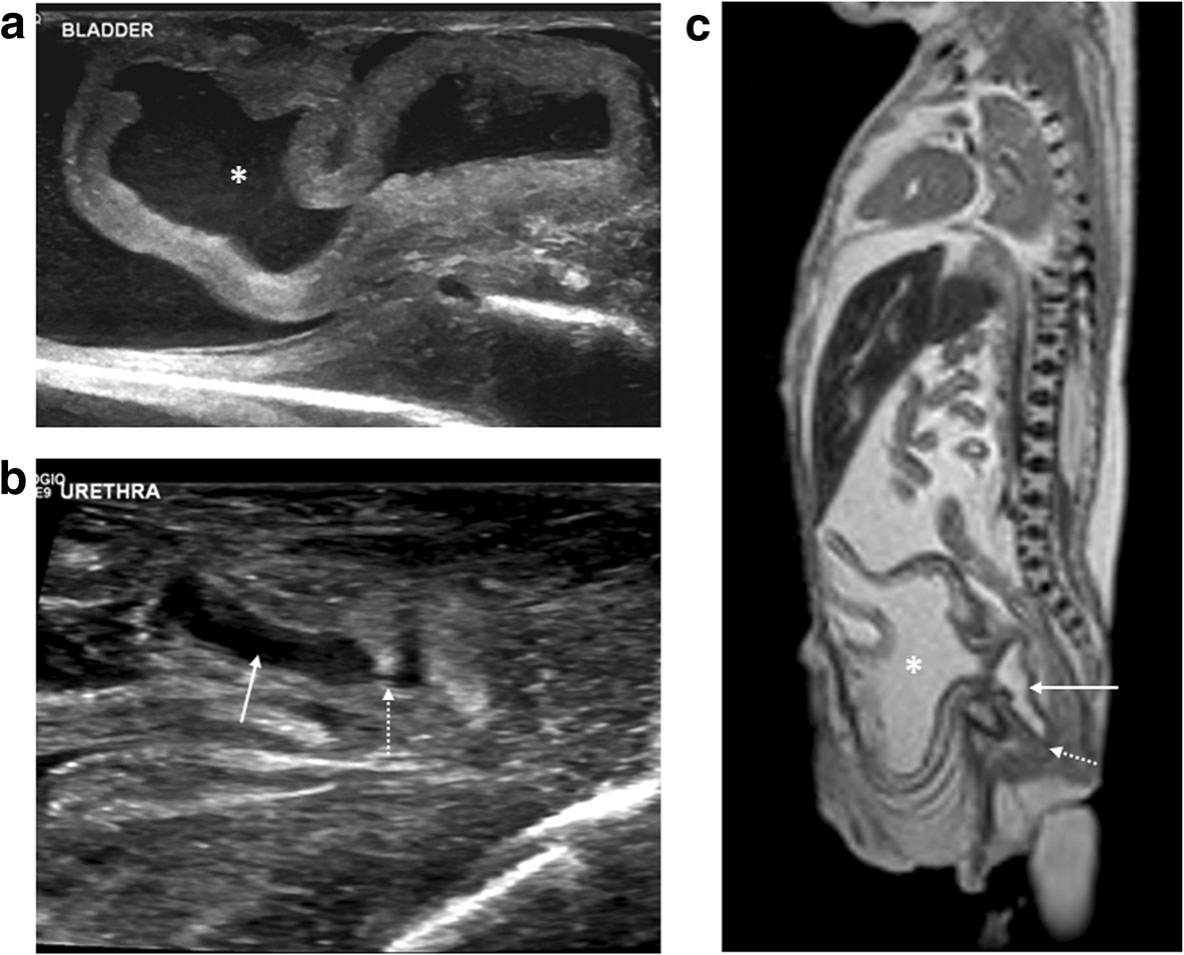
Bladder outlet obstruction due to posterior urethral valves in a foetus at 21 weeks gestation. The post-mortem imaging was obtained three days after termination of pregnancy. Sagittal post-mortem ultrasound images of the bladder (**a**) and posterior urethra (**b**) demonstrate a partially distended urinary bladder (asterisk) with thickened bladder wall, a dilated posterior urethra (solid arrow) and an abrupt urethral narrowing (dotted arrow) at site of obstruction. The corresponding sagittal post-mortem T2 weighted MRI study (**c**) also demonstrates the large urinary bladder (asterisk) with dilated posterior urethra (solid arrow), although the site of obstruction (dotted arrow) is less well depicted than on the ultrasound

**Fig. 18:**
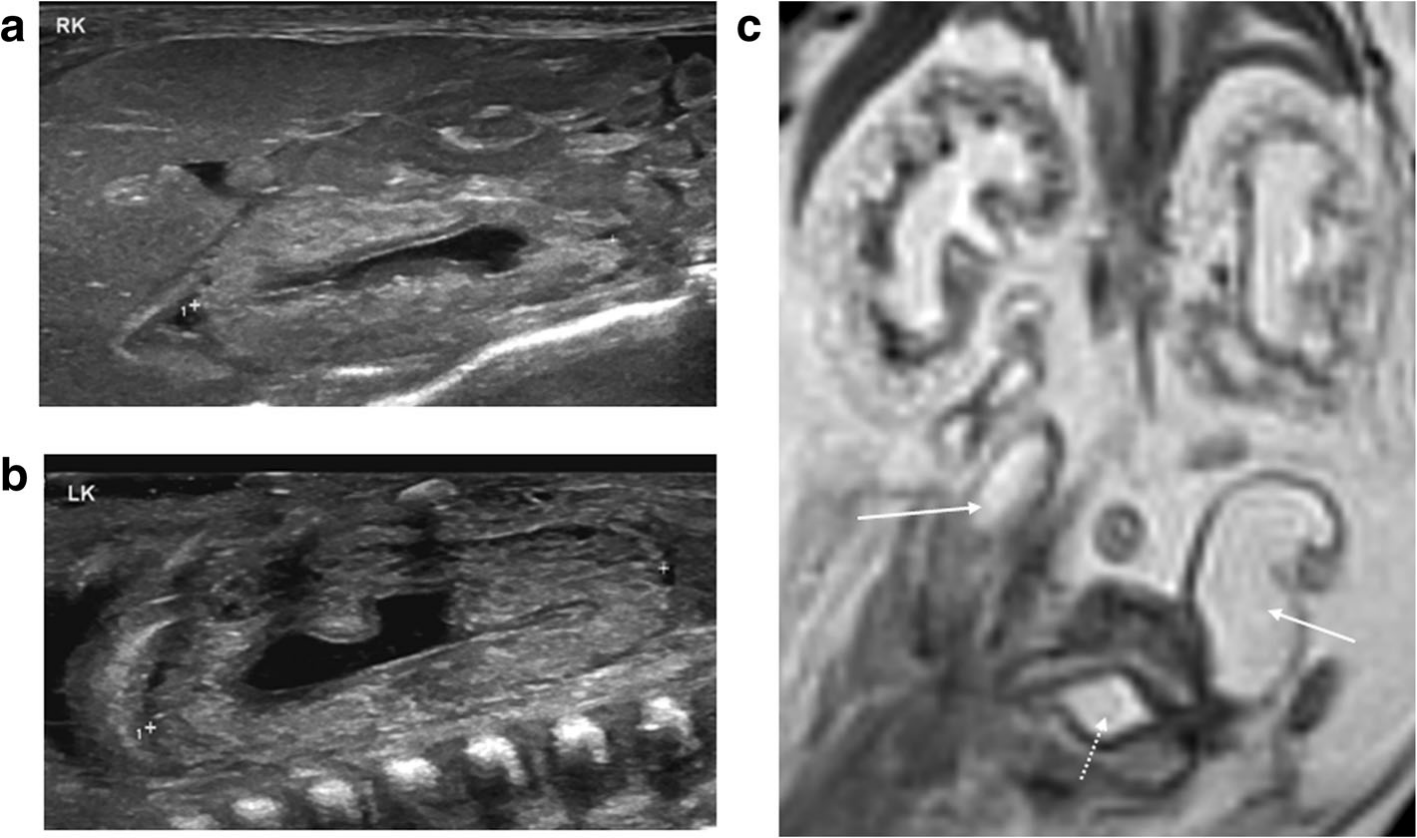
Dysplastic renal appearances in a foetus at 21 weeks gestation, seen as a result of bladder outflow obstruction. Post-mortem imaging was performed 4 days after termination of pregnancy. On the sagittal post-mortem ultrasound images of the right (**a**) and left (**b**) kidneys, there is loss of the normal corticomedullary differentiation with hyperechoic renal cortices and mild pelvicalyceal dilatation. The corresponding post-mortem T2 weighted MRI study in coronal plane (**c**) confirms the same findings as ultrasound, and also reveals bilateral dilated and tortuous ureters (solid arrows) leading to a partially collapsed urinary bladder (dotted arrow)

**Fig. 19:**
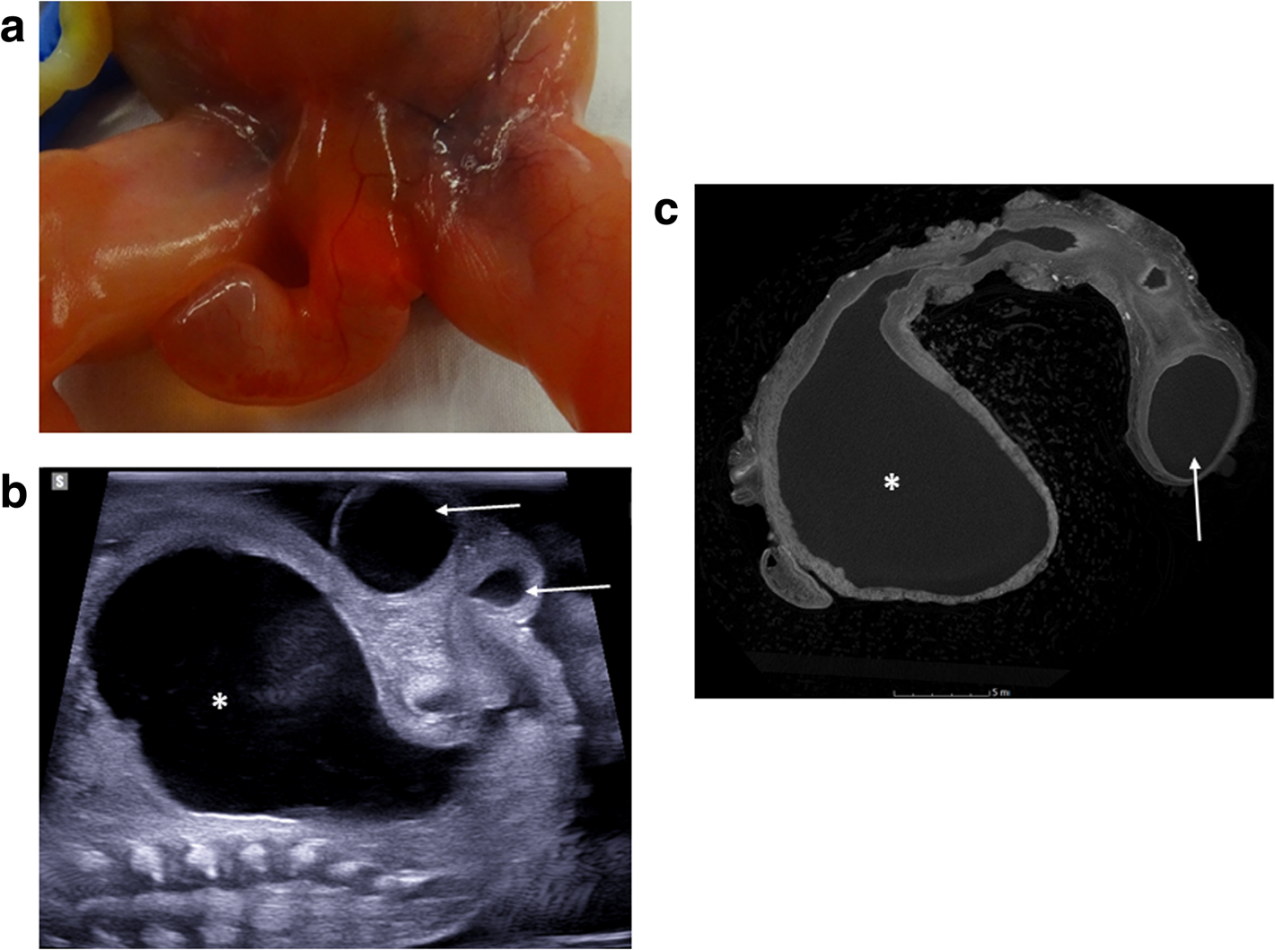
**a**–**c** Megalourethra in a foetus at 18 weeks gestation, following termination of pregnancy for bladder outlet obstruction and renal anomalies. A photograph of the perineum (**a**) obtained at external examination demonstrates an enlarged fluid-filled phallus without external meatus. Post-mortem ultrasound imaging of the pelvis in sagittal plane (**b**), acquired 6 days after delivery, reveals a distended urinary bladder (asterisk) with a dilated, fluid-filled and tortuous urethra (solid arrows). A subsequent micro-CT study (**c**) was performed of the extracted bladder and urethra prior to histological sectioning, demonstrating the same findings as the ultrasound of a dilated bladder and urethra. In other imaging planes (not shown), the corpus spongiosum was seen to be deficient in keeping with congenital scaphoid megalourethra

**Fig. 20:**
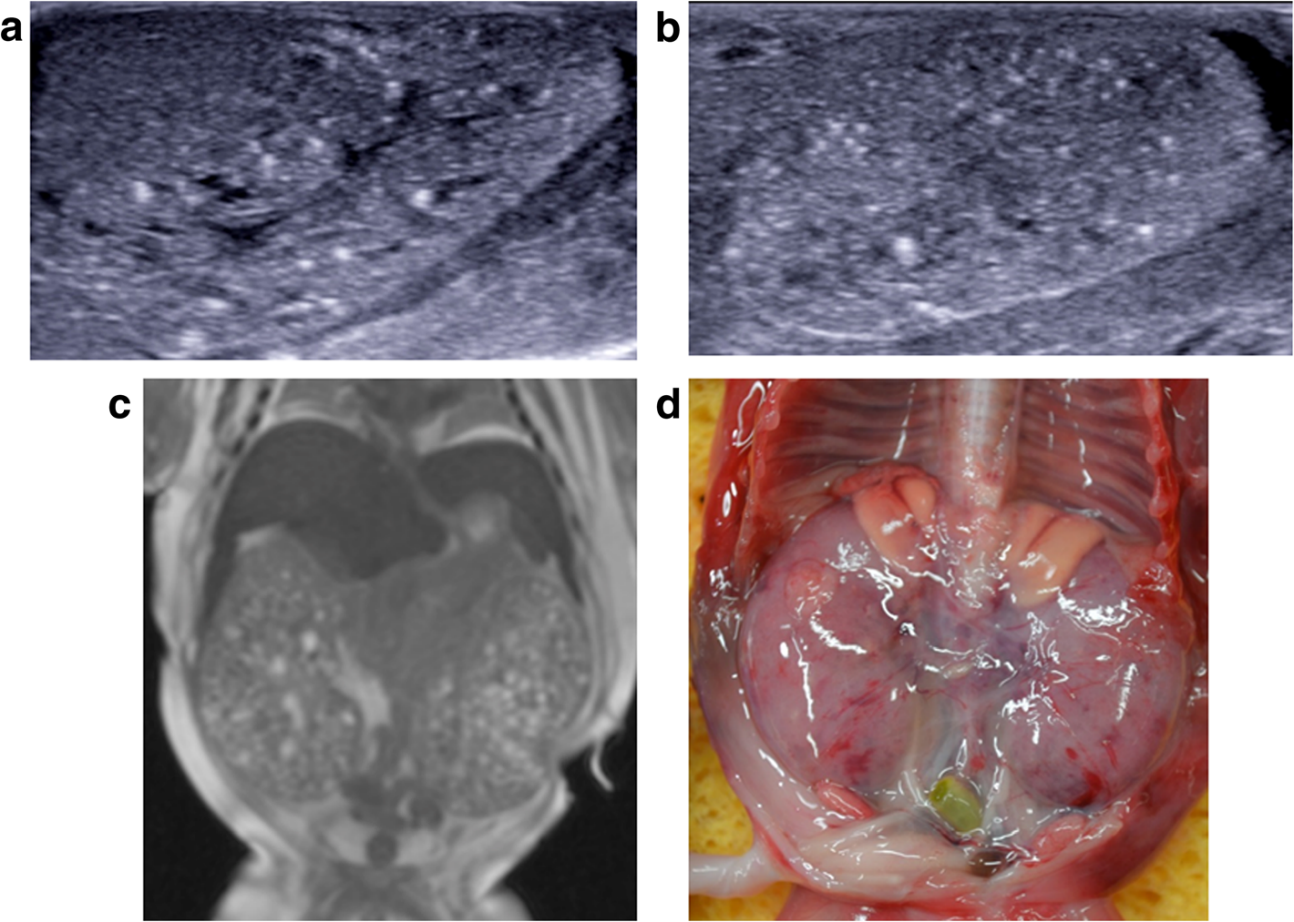
**a**–**c** Autosomal recessive polycystic kidney disease in a foetus at 17 weeks gestation, following termination of pregnancy. The post-mortem imaging was obtained 8 days after delivery. The sagittal post-mortem ultrasound images of the right (**a**) and left (**b**) kidneys demonstrate poor corticomedullary differentiation, numerous hyperechoic foci and a hyperechoic renal cortex in keeping with numerous renal microcysts. The corresponding post-mortem T2 weighted MRI in coronal plane (**c**), also shows enlarged kidneys with small microcysts and a normal appearing liver. At autopsy, the photograph taken of the retroperitoneum, with both kidneys in situ (**d**), shows the large size of these organs, occupying almost the entire abdomen

**Fig. 21:**
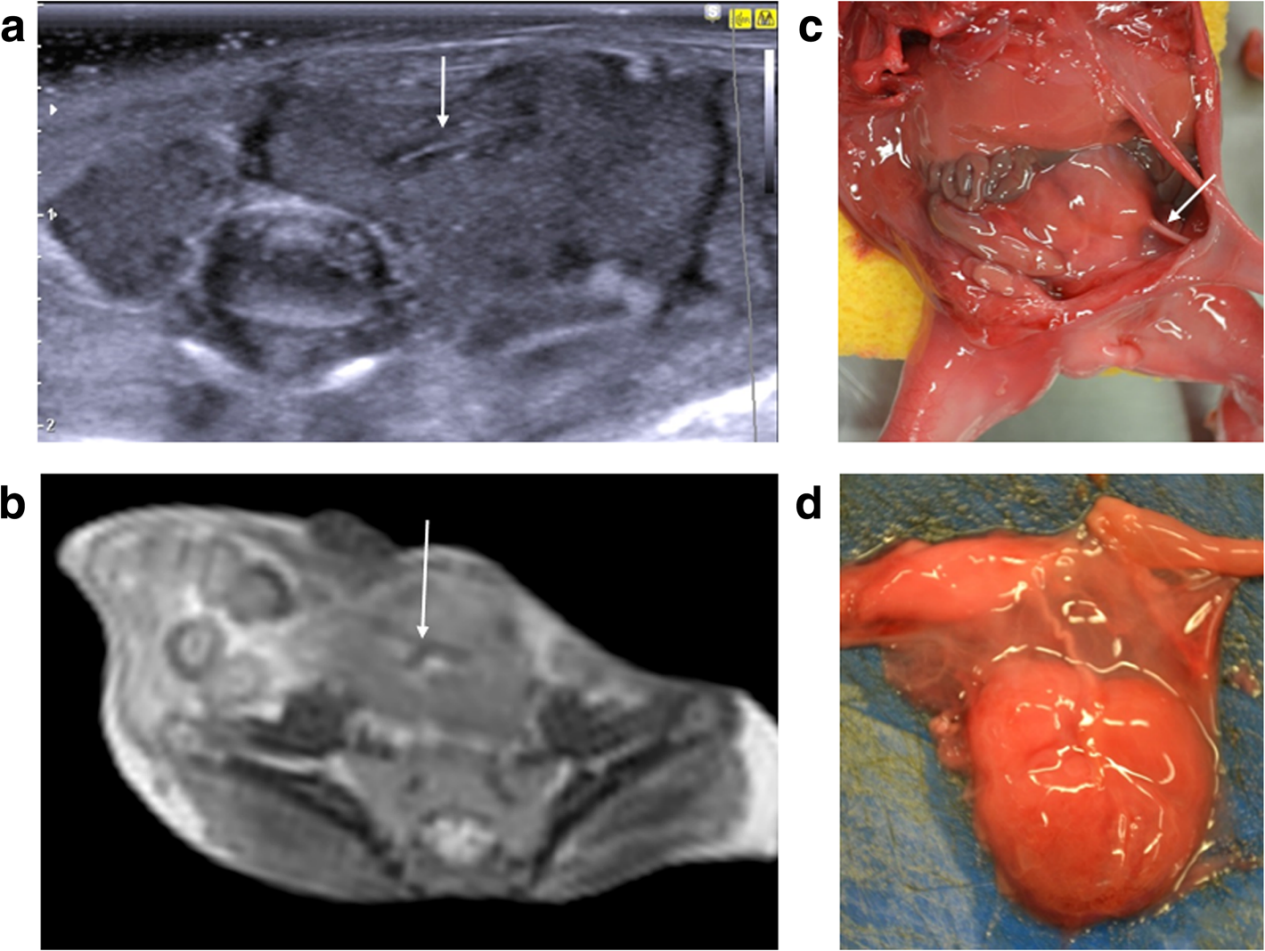
Solitary left pelvic kidney in a foetus at 20 weeks gestation, following termination of pregnancy for presumed renal aplasia. Post-mortem imaging was obtained 3 days after delivery. Transverse post-mortem ultrasound image (**a**) demonstrates a soft tissue mass in the left iliac fossa which lacks the normal expected renal corticomedullary differentiation but appears to have a demonstrable hilum (solid arrow). The same finding is demonstrated on the corresponding post-mortem T2 weighted MRI study, obtained in axial plane (**b**) and also on photographs obtained at autopsy (**c**), acquired after careful dissection of the anterior abdominal wall. The extracted soft tissue mass (**d**) was confirmed to be renal in origin

**Fig. 22:**
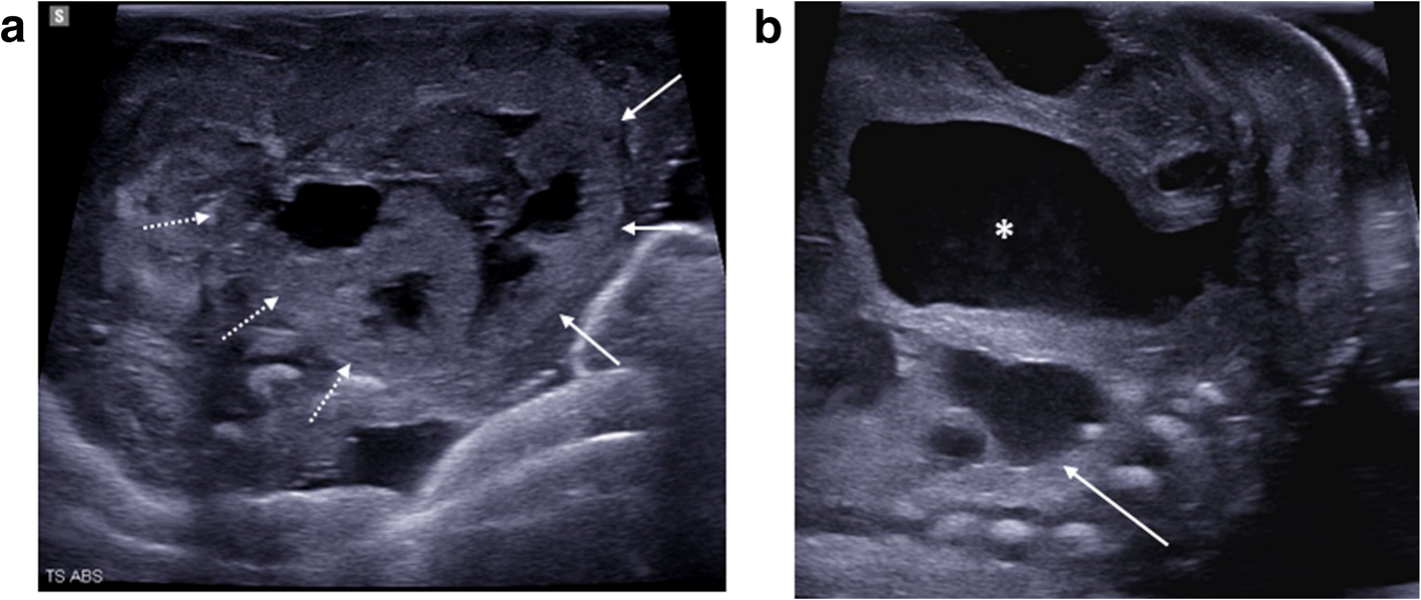
Cross-fused renal ectopia (‘pancake kidney’) in a foetus at 22 weeks gestation, following termination of pregnancy for multiple congenital anomalies. The post-mortem ultrasound images were acquired 10 days after delivery. Transverse ultrasound images through the lower abdomen (**a**) demonstrate cross-fused kidneys. The right moiety (dotted arrows) and left moiety (solid arrows) are demonstrated and show moderate calyceal dilation. On the sagittal ultrasound imaging of the distended urinary bladder (asterisk) (**b**), one can partially visualise the kidneys, demonstrating their pelvic location in the abdomen (solid arrow).

Renal anomalies are common and account for approximately 6–11% of all structural abnormalities seen at terminations of pregnancy [31, 45, 46]. These are usually related to renal dilatation (Figs. 16, 17, 18, and 19), polycystic renal disease (Fig. 20) or a mixture of structural congenital renal anomalies such as renal agenesis, ectopic kidneys (Fig. 21) or cross-fused ectopia (Fig. 22). A prenatal history of oligohydramnios may be present, and non-visualisation of the urinary bladder at prenatal ultrasound has been reported as a marker for significant renal pathology and poor foetal survival [47, 48].

### Musculoskeletal and soft tissue malformations

**Fig. 23:**
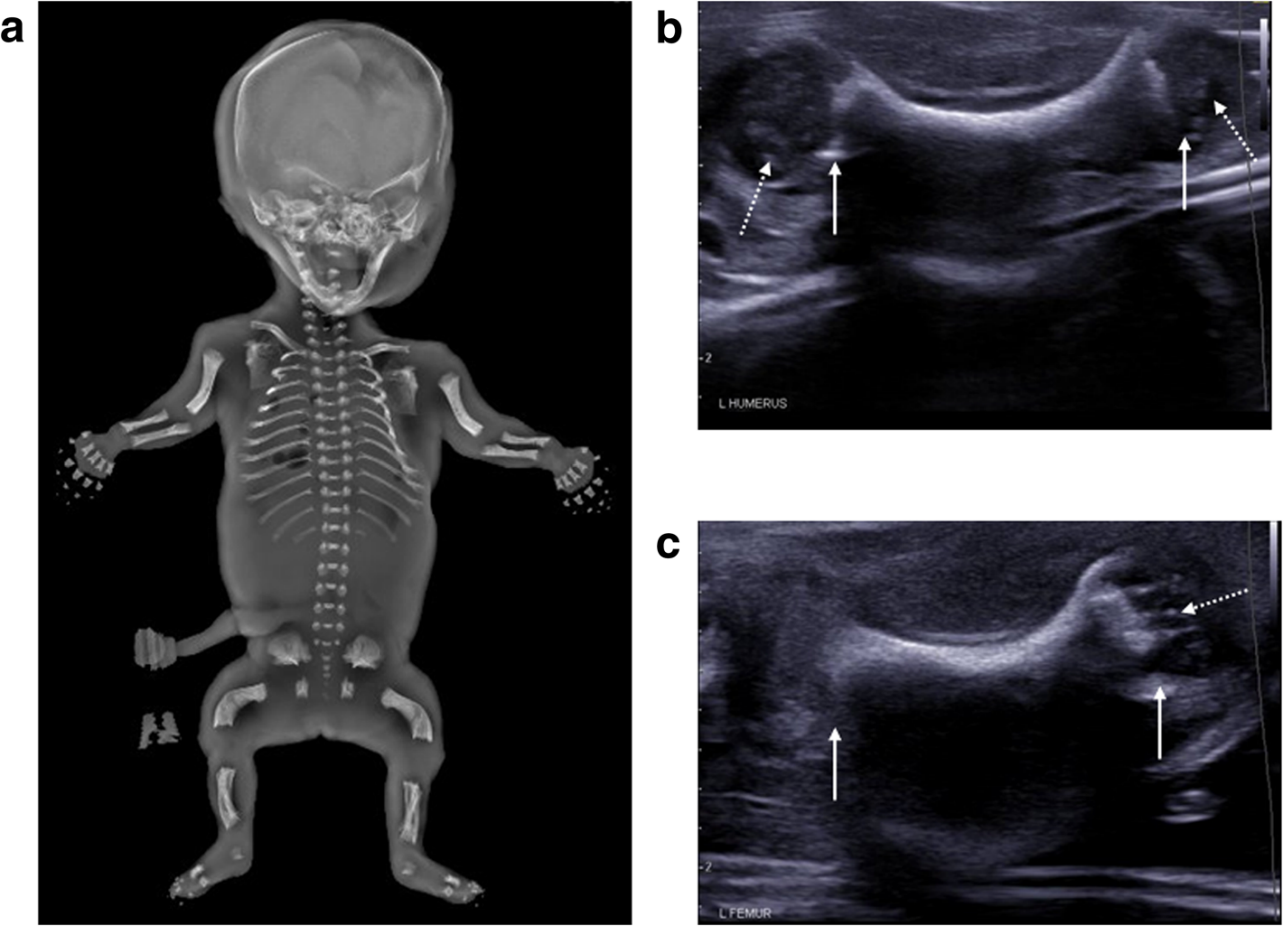
Thanatophoric dysplasia in a foetus at 22 weeks gestation, following termination of pregnancy. The frontal projection of the skeletal survey (**a**), acquired 2 days after delivery, demonstrates shortened long bones and curved femora with a trident appearance to the acetabulae. Post-mortem ultrasound images in sagittal plane of the left humerus (**b**) and left femur (**c**) reveal irregular metaphyses (solid arrows) and curved long bones. On ultrasound, it is also possible to visualise the unossified epiphyses (dotted arrows)

Where an underlying skeletal disorder is suspected, assessment of the whole skeleton is best performed by external examination and radiography (also known as a skeletal survey or babygram [32, 49, 50]). These are most useful over 8 weeks gestation, given the lack of skeletal ossification prior to this age [51]. It is also important to remember that although isolated limb anomalies may be more commonly encountered in live cases [52], lethal skeletal dysplasias will be more prevalent in the subgroup presenting for post-mortem imaging [53] so an entire overview of the body is usually necessary rather than imaging of the affected limb(s). Whilst PMUS can be useful in demonstrating the non-ossified, cartilaginous anatomy which is not present on radiography (Fig. 23), it is rarely used in isolation to make a skeletal diagnosis and would be better reserved for the assessment of soft tissues.

**Fig. 24:**
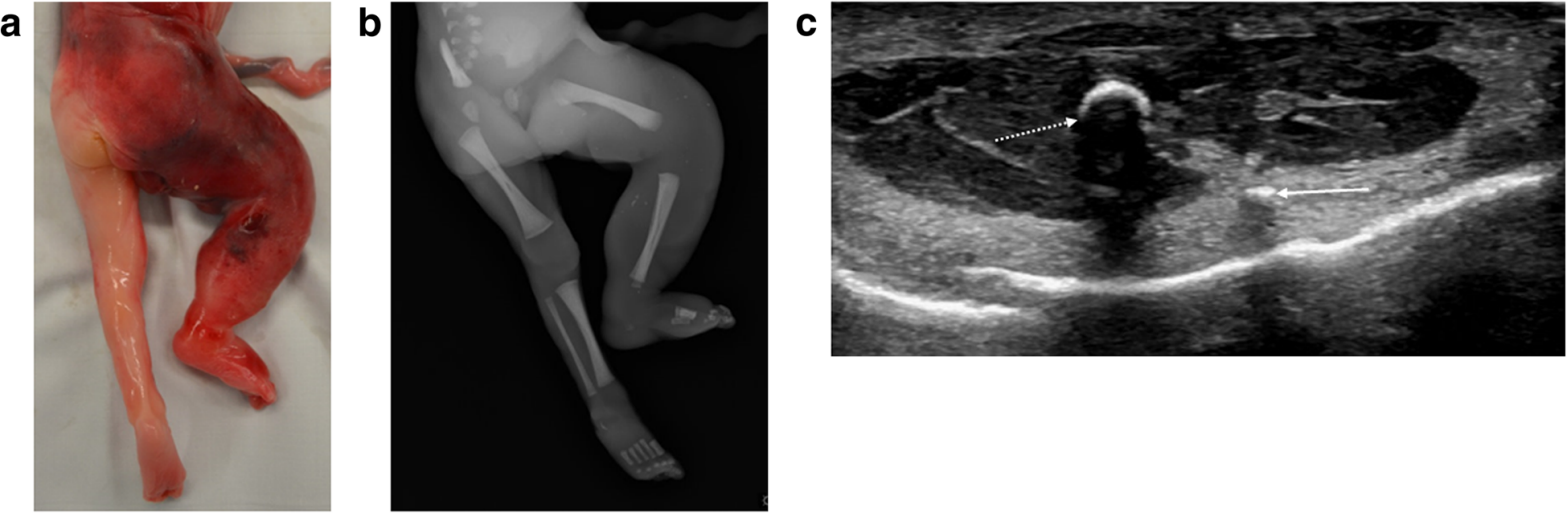
**a**–**c** Klippel-Trenaunay syndrome in a foetus at 22 weeks gestation, following termination of pregnancy. The external photograph of the lower limbs at autopsy (**a**), seen from behind, reveals an enlarged right leg with discoloured red and purple overlying skin. The post-mortem lateral radiograph of this right limb (**b**) reveals numerous small hyperdense foci within the soft tissues which are tiny phleboliths. At post-mortem ultrasound of the right thigh (**c**), obtained in axial plane 1 day after delivery, the small phleboliths were well seen with posterior acoustic shadowing. One of these is demonstrated with a solid arrow, the dotted arrow represents the right femur. At autopsy, extensive ectatic thin-walled vessels containing thrombus were seen in the right leg and in keeping with a vascular malformation. Due to lack of circulation and small diameter of vessels, this vascular anomaly could not be well depicted on ultrasound

Soft tissue lesions at PMUS may include venolymphatic (and other vascular) malformations (Fig. 24) or teratomas (as previously shown in Fig. 8) [44, 54]. In cases where a foetus is referred with a prenatal diagnosis of cystic hygroma (usually in the setting of foetal hydrops), we have also found that these cystic masses can ‘resolve’ [55], much like the appearances of ventriculomegaly, thus the role of PMUS is to review associated structural anomalies rather than identify the cystic hygroma itself. Associated anomalies (commonly intracranial in origin) are estimated to occur in 36–55.6% of cases, and usually related to an abnormal genetic karyotype [56, 57] which will have implications for prenatal counselling in future pregnancies [58].

## Future prospects

The future of perinatal post-mortem imaging has many opportunities for further research, particularly for evidence-based studies that allow healthcare professionals and parents to understand the additional clinical yield from different imaging examinations. Combining this scientific evidence with an educational, practical approach, whilst accounting for imaging availability and affordability, will inform the future imaging protocols in this field.

At present, there are no internationally agreed guidelines outlining appropriate referral indications for PMUS or documented perinatal post-mortem imaging pathway. Given that smaller, macerated foetuses are more likely to be non-diagnostic on PMUS and that cardiac anomalies are difficult to detect, one possible referral pathway may be in prioritising PMUS for larger (> 20 weeks gestation), non-macerated foetuses [28, 29] potentially alleviating the need for PMMR and to preferentially use PMMR for prenatally suspected cardiac anomalies. Alternatively, for clinical institutions where access to PMMR is more challenging, it may be more appropriate for all perinatal deaths to be offered a PMUS in the first instance, especially if parental consent for autopsy is refused and no other imaging alternatives exist.

Where tissue samples are required for genetic analysis or histopathological correlation, PMUS-guided needle biopsies could potentially be used to reduce the invasiveness of conventional autopsy techniques. ‘Blinded’ percutaneous needle biopsies using anatomical surface landmarks have been used with varying levels of success [59, 60], and CT-guided biopsies have also been proposed in the literature [61]. PMUS guidance may provide the optimal pragmatic solution enabling better organ visualisation (compared to ‘blinded biopsies’) and ease of access and affordability (compared with CT biopsies).

To our knowledge, there are no studies that have assessed the clinical change or additional contribution made by PMUS with respect to future pregnancy management, or whether there is any need for additional autopsy after comprehensive PMUS and prenatal imaging. More widespread use of the technique with pooling of data will allow these questions to be addressed.

## Conclusions

Perinatal post-mortem ultrasound is an easily accessible imaging tool that allows detailed visualisation of many common congenital pathologies. This article has highlighted several examples including ventriculomegaly, congenital diaphragmatic hernias and genitourinary malformations. Whilst there may be a higher yield of non-diagnostic images in smaller (earlier gestation) and macerated foetuses, concordance rates of ultrasound with autopsy remain high and there may be a role for this technique to serve as a ‘screening’ tool prior to post-mortem MRI or in aiding image-guided biopsies.

## Data Availability

Data sharing is not applicable to this article as no datasets were generated or analysed during the current study

## Abbreviations

CT: Computed tomography
MRI: Magnetic resonance imaging
PACS: Picture archiving and communications system
PMCT: Post-mortem computed tomography
PMMR: Post-mortem magnetic resonance imaging
PMUS: Post-mortem ultrasound

## References

[1] Horey D, Flenady V, Conway L, McLeod E, Yee Khong T (2014). Decision influences and aftermath: parents, stillbirth and autopsy. Health Expect.

[2] Heazell Alexander E P, Siassakos Dimitrios, Blencowe Hannah, Burden Christy, Bhutta Zulfiqar A, Cacciatore Joanne, Dang Nghia, Das Jai, Flenady Vicki, Gold Katherine J, Mensah Olivia K, Millum Joseph, Nuzum Daniel, O'Donoghue Keelin, Redshaw Maggie, Rizvi Arjumand, Roberts Tracy, Toyin Saraki H E, Storey Claire, Wojcieszek Aleena M, Downe Soo, Flenady Vicki, Frøen J Frederik, Kinney Mary V, de Bernis Luc, Lawn Joy E, Blencowe Hannah, Heazell Alexander E P, Leisher Susannah Hopkins, Radestad Ingela, Jackson Louise, Ogwulu Chidubem, Hills Alison, Bradley Stephanie, Taylor Wendy, Budd Jayne (2016). Stillbirths: economic and psychosocial consequences. The Lancet.

[3] Lewis C, Hill M, Arthurs OJ, Hutchinson C, Chitty LS, Sebire NJ (2018). Factors affecting uptake of postmortem examination in the prenatal, perinatal and paediatric setting. BJOG.

[4] Lewis C, Latif Z, Hill M, Riddington M, Lakhanpaul M, Arthurs OJ (2018). “We might get a lot more families who will agree”: Muslim and Jewish perspectives on less invasive perinatal and paediatric autopsy. PLoS One..

[5] Lewis C, Riddington M, Arthurs OJ (2019). Availability of less invasive prenatal, perinatal and paediatric autopsy will improve uptake rates: a mixed methods study with bereaved parents. BJOG..

[6] Ashwin C, Hutchinson JC, Kang X (2017). Learning effect on perinatal post-mortem magnetic resonance imaging reporting: single reporter diagnostic accuracy of 200 cases. Prenat Diagn.

[7] Arthurs OJ, Guy A, Thayyil S (2016). Comparison of diagnostic performance for perinatal and paediatric post-mortem imaging: CT versus MRI. Eur Radiol.

[8] Hutchinson John C., Kang Xin, Shelmerdine Susan Cheng, Segers Valerie, Lombardi Claudio M., Cannie Mieke M., Sebire Neil J., Jani Jacques C., Arthurs Owen J. (2018). Postmortem microfocus computed tomography for early gestation fetuses: a validation study against conventional autopsy. American Journal of Obstetrics and Gynecology.

[9] Shelmerdine SC, Sebire NJ, Arthurs OJ (2019). Perinatal post mortem ultrasound (PMUS): a practical approach. Insights Imaging.

[10] Rodriguez MA, Prats P, Rodriguez I, Cusi V, Comas C (2014). Concordance between prenatal ultrasound and autopsy findings in a tertiary center. Prenat Diagn.

[11] Struksnaes C, Blaas HG, Eik-Nes SH, Vogt C (2016). Correlation between prenatal ultrasound and postmortem findings in 1029 fetuses following termination of pregnancy. Ultrasound Obstet Gynecol.

[12] Rossi AC, Prefumo F (2017). Correlation between fetal autopsy and prenatal diagnosis by ultrasound: a systematic review. Eur J Obstet Gynecol Reprod Biol.

[13] Debost-Legrand A, Laurichesse-Delmas H, Francannet C, Lemery ID, Gallot D, Venditelli F (2014). False positive morphologic diagnoses at the anomaly scan: marginal or real problem, a population-based cohort study. BMC Pregnancy Childbirth.

[14] Ozyuncu O, Orgul G, Tanacan A (2019). Retrospective analysis of indications for termination of pregnancy. J Obstet Gynaecol.

[15] Monier I, Lelong N, Ancel PY (2019). Indications leading to termination of pregnancy between 22(+0) and 31(+6) weeks of gestational age in France: a population-based cohort study. Eur J Obstet Gynecol Reprod Biol.

[16] Man J, Hutchinson JC, Heazell AE, Ashworth M, Levine S, Sebire NJ (2016). Stillbirth and intrauterine fetal death: factors affecting determination of cause of death at autopsy. Ultrasound Obstet Gynecol.

[17] Kang X, Cos T, Guizani M, Cannie MM, Segers V, Jani JC (2014). Parental acceptance of minimally invasive fetal and neonatal autopsy compared with conventional autopsy. Prenat Diagn.

[18] Lewis C, Hill M, Arthurs OJ, Hutchinson JC, Chitty LS, Sebire N (2018). Health professionals’ and coroners’ views on less invasive perinatal and paediatric autopsy: a qualitative study. Arch Dis Child.

[19] Arthurs OJ, Barber JL, Taylor AM, Sebire NJ (2015). Normal perinatal and paediatric postmortem magnetic resonance imaging appearances. Pediatr Radiol.

[20] Arthurs OJ, Taylor AM, Sebire NJ (2015). Indications, advantages and limitations of perinatal postmortem imaging in clinical practice. Pediatr Radiol.

[21] Arthurs OJ, Thayyil S, Addison S (2014). Diagnostic accuracy of postmortem MRI for musculoskeletal abnormalities in fetuses and children. Prenat Diagn.

[22] Arthurs OJ, Thayyil S, Olsen OE (2014). Diagnostic accuracy of post-mortem MRI for thoracic abnormalities in fetuses and children. Eur Radiol.

[23] Arthurs OJ, Thayyil S, Owens CM (2015). Diagnostic accuracy of post mortem MRI for abdominal abnormalities in foetuses and children. Eur J Radiol.

[24] Arthurs OJ, Thayyil S, Pauliah SS (2015). Diagnostic accuracy and limitations of post-mortem MRI for neurological abnormalities in fetuses and children. Clin Radiol.

[25] Kang X, Shelmerdine SC, Hurtado I (2019). Postmortem examination of human fetuses: comparison of two-dimensional ultrasound with invasive autopsy. Ultrasound Obstet Gynecol..

[26] Tuchtan L., Lesieur E., Bartoli C., Delteil C., Sarda-Quarello L., Torrents J., Sigaudy S., Piercecchi M.-D., Gorincour G. (2018). Diagnosis of congenital abnormalities with post-mortem ultrasound in perinatal death. Diagnostic and Interventional Imaging.

[27] Prodhomme O, Baud C, Saguintaah M, Béchard-Sevette N, Bolivar J, David S, Taleb-Arrada I, Couture A (2015). Comparison of postmortem ultrasound and X-Ray with autopsy in fetal death: retrospective study of 169 cases. Journal of Forensic Radiology and Imaging.

[28] Kang X, Cos Sanchez T, Arthurs OJ et al (2019) Postmortem fetal imaging: a prospective blinded comparison study of 2-dimensional ultrasound with MR imaging. Ultrasound Obstet Gynecol. 10.1002/uog.20217 Epub ahead of print 10.1002/uog.20217 30644623

[29] Cain MA, Guidi CB, Steffensen T, Whiteman VE, Gilbert-Barness E, Johnson DR (2014). Postmortem ultrasonography of the macerated fetus complements autopsy following in utero fetal demise. Pediatr Dev Pathol.

[30] Arslan E, Buyukkurt S, Sucu M (2018). Detection of major anomalies during the first and early second trimester: Single-center results of six years. J Turk Ger Gynecol. Assoc.

[31] Hern WM (2014). Fetal diagnostic indications for second and third trimester outpatient pregnancy termination. Prenat Diagn.

[32] Arthurs OJ, Calder AD, Kiho L, Taylor AM, Sebire NJ (2014). Routine perinatal and paediatric post-mortem radiography: detection rates and implications for practice. Pediatr Radiol.

[33] Sebire NJ, Miller S, Jacques TS (2013). Post-mortem apparent resolution of fetal ventriculomegaly: evidence from magnetic resonance imaging. Prenat Diagn.

[34] Edwards L, Hui L (2018). First and second trimester screening for fetal structural anomalies. Semin Fetal Neonatal Med.

[35] Robles Fradejas M., Gonzalo García I., De las Casas Quispe A. C., Martin García A., García Higuera M. I., Rodriguez Minguélez M., Martínez-Guisasola J. (2016). Fetal intracranial immature teratoma: presentation of a case and a systematic review of the literature. The Journal of Maternal-Fetal & Neonatal Medicine.

[36] Milani HJ, Araujo Junior E, Cavalheiro S (2015). Fetal brain tumors: prenatal diagnosis by ultrasound and magnetic resonance imaging. World J Radiol.

[37] Garel C, Moutard ML (2014). Main congenital cerebral anomalies: how prenatal imaging aids counseling. Fetal Diagn Ther.

[38] Morris JK, Springett AL, Greenlees R (2018). Trends in congenital anomalies in Europe from 1980 to 2012. PLoS One.

[39] Shelmerdine SC, Hickson M, Sebire NJ, Arthurs OJ (2019). Post-mortem magnetic resonance imaging appearances of feticide in perinatal deaths. Fetal Diagn Ther.

[40] Vincenti M, Guillaumont S, Clarivet B (2019). Prognosis of severe congenital heart diseases: do we overestimate the impact of prenatal diagnosis?. Arch Cardiovasc Dis..

[41] Fowler DJ, Gould SJ (2015). The pathology of congenital lung lesions. Semin Pediatr Surg.

[42] Durell J, Lakhoo K (2014). Congenital cystic lesions of the lung. Early Hum Dev.

[43] Votino C, Cos Sanchez T, Bessieres B (2018). Minimally invasive fetal autopsy using ultrasound: a feasibility study. Ultrasound Obstet Gynecol.

[44] Masmejan S, Baud D, Ryan G, Van Mieghem T (2019) Management of fetal tumors. Best Pract Res Clin Obstet Gynaecol. Pii: S1521-6934(18)30201-3 [Epub ahead of print] 10.1016/j.bpobgyn.2019.01.006 30770283

[45] Vaknin Z, Lahat Y, Barel O (2009). Termination of pregnancy due to fetal abnormalities performed after 23 weeks’ gestation: analysis of indications in 144 cases from a single medical center. Fetal Diagn Ther.

[46] Barel O, Vaknin Z, Smorgick N (2009). Fetal abnormalities leading to third trimester abortion: nine-year experience from a single medical center. Prenat Diagn.

[47] Simoens E, Hindryckx A, Moerman P, Claus F, De Catte L (2015). Termination of pregnancy for renal malformations. Pediatr Nephrol.

[48] Kumari N, Pradhan M, Shankar VH, Krishnani N, Phadke SR (2008). Post-mortem examination of prenatally diagnosed fatal renal malformation. J Perinatol.

[49] Kamphuis-van Ulzen K, Koopmanschap DH, Marcelis CL, van Vugt JM, Klein WM (2016). When is a post-mortem skeletal survey of the fetus indicated, and when not?. J Matern Fetal Neonatal Med.

[50] Olsen EO, Espeland A, Maartmann-Moe H, Lachman RS, Rosendahl K (2003). Diagnostic value of radiography in cases of perinatal death: a population based study. Arch Dis Child Fetal Neonatal Ed.

[51] Calder AD, Offiah AC (2015). Foetal radiography for suspected skeletal dysplasia: technique, normal appearances, diagnostic approach. Pediatr Radiol.

[52] Bedard T, Lowry RB, Sibbald B, Kiefer GN, Metcalfe A (2015). Congenital limb deficiencies in Alberta-a review of 33 years (1980-2012) from the Alberta Congenital Anomalies Surveillance System (ACASS). Am J Med Genet A.

[53] Offiah AC (2015). Skeletal Dysplasias: An Overview. Endocr Dev..

[54] Papadopoulou I, Sebire NJ, Shelmerdine SC, Bower S, Arthurs OJ (2015). Postmortem image-guided biopsy for less-invasive diagnosis of congenital intracranial teratoma. Ultrasound Obstet Gynecol.

[55] Noia G, Pellegrino M, Masini L (2013). Fetal cystic hygroma: the importance of natural history. Eur J Obstet Gynecol Reprod Biol.

[56] Grapsa D, Mavrigiannaki P, Kleanthis C, Hasiakos D, Vitoratos N, Kondi-Pafiti A (2012). Autopsy findings in fetuses with cystic hygroma: a literature review and our center’s experience. Clin Exp Obstet Gynecol.

[57] Scholl J, Chasen ST (2016). First trimester cystic hygroma: does early detection matter?. Prenat Diagn.

[58] Chen CP, Liu FF, Jan SW, Lee CC, Town DD, Lan CC (1996). Cytogenetic evaluation of cystic hygroma associated with hydrops fetalis, oligohydramnios or intrauterine fetal death: the roles of amniocentesis, postmortem chorionic villus sampling and cystic hygroma paracentesis. Acta Obstet Gynecol Scand.

[59] Breeze AC, Jessop FA, Whitehead AL (2008). Feasibility of percutaneous organ biopsy as part of a minimally invasive perinatal autopsy. Virchows Arch.

[60] Menendez C, Castillo P, Martinez MJ (2017). Validity of a minimally invasive autopsy for cause of death determination in stillborn babies and neonates in Mozambique: an observational study. PLoS Med.

[61] Ruegger CM, Bartsch C, Martinez MJ (2014). Minimally invasive, imaging guided virtual autopsy compared to conventional autopsy in foetal, newborn and infant cases: study protocol for the paediatric virtual autopsy trial. BMC Pediatr..

